# Evaluating the acceptability and feasibility of new mosquito bite prevention tools in a “forest pack” to support malaria elimination in Cambodia

**DOI:** 10.1186/s12936-025-05682-2

**Published:** 2025-11-27

**Authors:** David J. McIver, Dyna Doum, Chean Men, Josselyn Neukom, Neil F. Lobo, Jason H. Richardson, Michael Macdonald, Priya B. Shete, Siv Sovannaroth, Allison Tatarsky

**Affiliations:** 1 Malaria Elimination Initiative, Institute for Global Health Sciences, University of California, San Francisco, 550 16th Street, San Francisco, CA 94158 USA; 2 Health Forefront Organization, Phnom Penh, Cambodia; 3 Eck Institute for Global Health, University of Notre Dame, South Bend, IN 46556 USA; 4 Innovative Vector Control Consortium, Liverpool, UK; 5 Center for Tuberculosis, Institute for Global Health Sciences, University of California, San Francisco, 550 16th Street, San Francisco, CA 94158 USA; 6 National Center for Parasitology, Entomology and Malaria Control, 477, Phnom Penh, Cambodia

## Abstract

**Background:**

Cambodia is nearing malaria elimination after years of sustained progress. The remaining challenges are at-risk populations living or working in forested areas exposed to outdoor and early evening biting and where treated nets or residual spraying is not practical. This study evaluates the acceptability and feasibility of additional mosquito bite protection products, delivered to these high-risk populations.

**Methods:**

This study was conducted in two high-malaria burden operational districts (OD) in Cambodia, targeting high-risk individuals: forest goers, forest dwellers, and forest rangers. A total of 28,000 forest packs containing a volatile pyrethroid spatial repellent (VPSR) and topical repellent (TR), were distributed over four rounds and clothing was treated with insecticide over two rounds. Social and Behavior Change Communication (SBCC) materials to support adoption were included. The evaluation included surveys, key informant interviews, and focus group discussions. Outcomes were assessed using implementation science frameworks and descriptive statistics.

**Results:**

A total of 2935 individuals from 18 villages were enrolled. Product distribution through village malaria workers reached 85% of the target population in Sen Monorom OD and between 72–102% in Phnom Srouch OD, though pack fidelity varied, with fewer complete packs delivered than planned, particularly in Phnom Srouch. Coverage of ITC was very low across both ODs (1–40% in Sen Monorom and 1–5% in Phnom Srouch). Reported acceptability and appropriateness of the products increased over time, with up to 96% of participants finding the SE acceptable, 93% for TR, and 100% for insecticide treated clothing (ITC). Reported product use was high, particularly among forest dwellers; forest rangers had lower reported use, especially for topical repellent. Users indicated the potential for early and consistent SBCC engagement to improve use. Having trusted members of the community deliver tools and SBCC is important. Each tool was reported to be most useful in different use scenarios and at different times of the day. Government and NGO implementers believed the products were appropriate for the targeted groups and that, while delivery to remote communities was challenging, especially for ITC, they support implementation of these products for malaria elimination.

**Conclusion:**

This study demonstrated the acceptability and feasibility of new bite prevention products distributed together with SBCC, from the perspective of forest-exposed, at-risk community members. It highlighted the need for better implementation to ensure more timely and consistent dissemination of product and SBCC materials. Further studies are needed to assess the epidemiological impact of combined bite prevention tools.

**Supplementary Information:**

The online version contains supplementary material available at 10.1186/s12936-025-05682-2.

## Background

Cambodia is targeting the elimination of all human malaria by 2025 [1], reporting just 355 confirmed cases in 2024, 85% of them *Plasmodium vivax* [2]. As in other Greater Mekong Subregion (GMS) countries, the remaining malaria risk in Cambodia is concentrated among people living or working in forested areas with limited health service access and persistent exposure to mosquito vectors indoors and outdoors [3]. The primary malaria prevention interventions currently in use in these populations include insecticide treated bed nets and hammock nets (ITNs and ITHNs), chemoprophylaxis, and, in some cases, topical repellents [4, 5]. To reach malaria elimination in this last mile, additional bite prevention tools are necessary for these populations to further reduce exposure to potentially infective bites.

Project BITE (Bite Interruption Toward Elimination) is a research programme designed to evaluate the efficacy and acceptability of novel vector control tools for use in the GMS and beyond. The programme evaluated individual tools as well as the combination of tools into a “forest pack”, for individuals at high risk of malaria. Although national malaria programmes in the GMS have distributed earlier versions of forest packs—including ITNs, ITHNs, and rubber boots—these did not fully address remaining gaps in protection [5, 6]. Prior research from Project BITE, including both semi-field system studies [7] and entomological field studies [8], have demonstrated that three vector control tools—volatile pyrethroid spatial repellents (VPSRs), insecticide treated clothing (ITC) (including with an etofenprox solution), and topical repellents (TR)—are efficacious at preventing mosquito landing and also led to increased knock-down, delayed blood feeding, and increased mortality of mosquitoes, indicating potential for community impact. Modelling studies also predict that these tools, with effective targeting and sufficient coverage, may substantially reduce vectorial capacity and the entomological inoculation rate as a proxy for transmission [9]. Formative work [10] described preliminary acceptability and feasibility of implementation of these three tools, and entomological assessments revealed gaps in protection at various spaces and times where and when new tools could offer protection [11]. Economic studies demonstrate that the potential costs [12] and willingness-to-pay threshold [13] were favourable for large-scale implementation depending on the final price of these novel products for the public health sector.

This final study of the BITE research programme aimed to evaluate the implementation of a new forest pack containing these same three products: a VPSR, insecticidal treatment of clothing, and topical repellent, supported by Social and Behaviour Change Communication (SBCC) and distribution targeting individuals at risk. The success of these bite prevention tools depends on their appropriateness and acceptability to end users and the feasibility and sustainability of implementation for informing programmatic use and scale-up [14–18].

## Methods

### Target populations and study sites

This was an implementation feasibility study, with objectives to understand the feasibility of delivering a forest pack containing novel bite prevention tools to a large population, and to learn how end-users felt about and interacted with those tools. Forest packs were delivered to individuals determined to be at high risk of malaria infection due to forest exposure, who were characterized as:

1. Forest goers: people who live one kilometre or more from the forest, but who travel to or through the forest regularly for purposes related to work (incl. farming, hunting, foraging, logging) or movement (travelling from one village to another)
2. Forest dwellers: people who live inside the forest or within one kilometer from the forest edge. Forest dwellers may have a permanent house in non-forested areas for part of the year, but move inside the forest during other parts of the year for seasonal harvests.
3. Forest rangers: people who are employed through the government or NGOs to protect border regions, patrol protected forests and wildlife areas, and who travel in the forests for this work for extended periods (at least 10 days per month)

The two sites chosen for this implementation feasibility study, Sen Monorom Operational District (OD) in Mondulkiri province and Phnom Srouch OD in Kampong Speu province, were selected in consultation with the Cambodia National Centre for Parasitology, Entomology, and Malaria Control (CNM) for three reasons: (1) they were among the highest malaria burden ODs in Cambodia at the time of site selection in 2022, (2) they are locations where the primary malaria risk factor is exposure to forest-associated vectors, and (3) these ODs were included in other aspects of the larger Project BITE research programme. Forest pack delivery in Sen Monorom OD was managed by Malaria Consortium, a not-for-profit organization, and pack delivery in Phnom Srouch OD was managed by CNM. Both distributions were conducted primarily through village malaria workers. Further details about study sites and target groups are listed in Table 1.

**Table 1:** Study site characteristics

Study site OD	Study site province	Landscape	2022 malaria cases*	Dominant malaria vectors**	Target populations
Sen Monorom	Mondulkiri	Large swaths of forests which are used by local populations for hunting and subsistence farming	494 (21.5% *P. falciparum*)	Subgroup Leucosphyrus species, *An. dirus*, and *An. maculatus*	Forest goers, forest dwellers, forest rangers
Phnom Srouch	Kampong Speu	Rapidly decreasing areas of forests which are being converted to farming or industrial uses	232 (5.8% *P. falciparum*)	Subgroup Leucosphyrus species, *An. dirus*, and *An. peditaeniatus*	Forest goers, forest dwellers

OD: Operational District; An: *Anophele*s

^*^data from Cambodia National Malaria Information System (MIS) at the time of study planning

^**^data from[11]

### Forest pack bite prevention products and distribution frequency

**Fig. 1:**
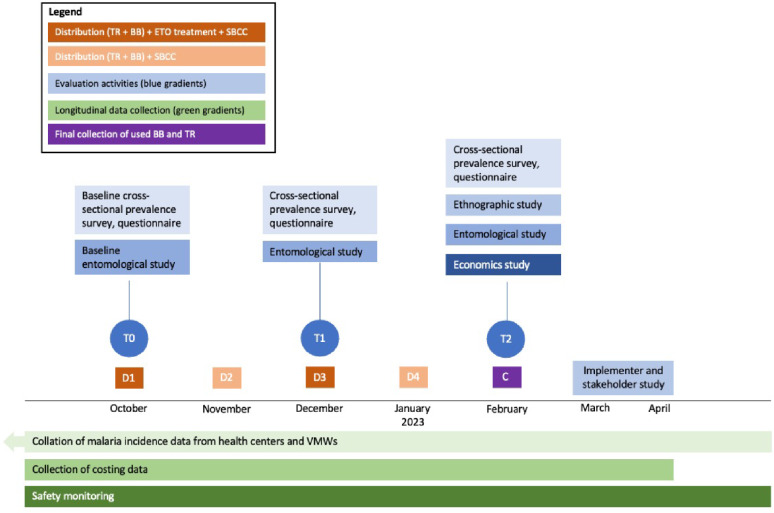
Overview of study timeline, including evaluation timepoints (T0-T2), distribution points (D1-D4), and data collected at each. BB: BiteBarrier (volatile pyrethroid spatial repellent); ETO: etofenprox treated clothing; TR: topical repellent

Based on the number of forest pack products available for this implementation feasibility study, the project aimed to deliver forest packs to 3500 individuals, in each province, at each of four distribution time points (D1–D4, each one month apart) (Fig. 1), for a total of 28,000 packs. Distribution 1 and Distribution 3 contained all three products—picaridin topical repellent, a transfluthrin spatial repellent, and etofenprox solution for clothing treatment—while Distribution 2 and Distribution 4 excluded the etofenprox solution (Fig. 1). Details of forest pack bite prevention tools are presented in Table 2. Etofenprox-treated clothing remains effective against mosquitoes for up to 25 washes and, therefore, did not require re-treatment at D2 and D4.SBCC materials were provided with each distribution, either as a leaflet for the user or as an interactive engagement tool designed to be used by the distributor to discuss the benefits of the tools and appropriate use with individuals at risk (Appendix 1). In both ODs, local health facility staff, public health department staff, and village malaria workers (VMWs) led the delivery of tools and SBCC in partnership with the lead implementing agency. Detailed records were kept of the number of packs delivered in each village, and whether the packs were complete or not (i.e. contained all three bite prevention tools at D1 and D3 and just the VPSR and topical repellent at D2 and D4).

**Table 2:** Project BITE Forest Pack Contents

Forest pack tool	Product name (manufacturer)	Active ingredient (pyrethroid class insecticide)	Description
Volatile Pyrethroid Spatial Repellent	BiteBarrier (PIC Corporation)	Transfluthrin	Approximately the size of an A4 piece of paper, impregnated with transfluthrin which passively emanates the active ingredient to provide protection in the surrounding area. This tool was included in the physical forest pack
Topical Repellent	Autan Topical Repellent (SC Johnson & Son Pty Ltd)	Picaridin	An aerosol topical repellent containing 20% picaridin, to be applied to exposed skin. This tool was included in the physical forest pack
Insecticide Treated Clothing	Perimeter ETO Insect Guard formulation (Pine Belt Processing Inc., a wholly owned subsidiary of Warmkraft, Inc.)	Etofenprox	An etofenprox-based, concentrated liquid solution for the self-treatment of clothing. Concentrated solution is mixed with water and applied to clothing using a hand-held sprayer. Village malaria workers applied the solution to participants’ clothing using the sprayer. At D1, clothing was treated at a central location in the village. By D3, delivery had evolved so that most people had their clothing treated by VMWs at their own home
Social and Behavioural Change Communication materials	SBCC materials took a different form at each of four distribution points D1 • A “how-to” document (leaflet) in Khmer instructing users on how to properly and effectively use each product • Included within the forest pack D2 • A discussion guide in Khmer designed to elicit a discussion between the end user and the VMW providing the forest pack • Provided to VMWs (or other personnel delivering forest packs) to engage directly with forest pack recipients D3 • An illustrated wall-hanging with Khmer messaging was designed to be hung in the users’ household, as a visual reminder to use the forest pack tools whenever possible • Included within the forest pack D4 • A video animation (with Khmer language audio) describing all the forest pack tools in detail, how and when to use them • Animation was also viewed and discussed during training for VMWs and health facility staff • Provided to VMWs (or other personnel delivering forest packs) through smart phone or tablet devices to engage directly with forest pack recipients • A “hanging reminder” poster in Khmer was also provided at D4, which instructed users to remember to use all three tools and to ask VMWs if they have any questions • Included within the forest pack • The Bunong language is also used by some people in Sen Monorom, though the written language was only recently developed and consequently very few Bunong speakers can read or write the language. Where appropriate, materials were translated orally by distributors for Bunong speakers who could not also speak or read Khmer		

A *complete pack* is defines as containing all products intended at that distribution round (VPSR, TR, and ETO at D1 and D3; VPSR and TR at D2 and D4). A *partial pack* refers to any pack missing one or more of the intended products. In practice, partial packs almost always reflected absence of ETO at D1 or D3, since VPSR and TR were nearly universally included at all rounds.

### Study timeline

Forest packs were distributed at one-month intervals for four consecutive months (D1-D4). Research activities overlapped with implementation, including cross-sectional surveys at three time points. Timepoint 0 (T0) occurred in October 2022, before any forest packs had been delivered, Timepoint 1 (T1) occurred in December 2022 following Distribution 3, and Timepoint 2 (T2) occurred in February 2023 following Distribution 4. Each Timepoint consisted of questionnaire administration and blood collection from all participants, as well as entomological collections. During T2, key informant interviews and focus groups were held with both study participants and implementation partners. Economic studies on the cost of delivering forest packs and willingness to pay for forest pack tools were also conducted during T2, with results reported elsewhere [12, 13]. Results from the parasite prevalence surveys and entomological collections conducted across T0–T2 are reported elsewhere [11, 19].

### Data collection procedures and analysis

#### Forest pack distribution

Distribution metrics related to the distribution of forest packs were collected using paper forms in the field during D1-D4 by both Malaria Consortium and CNM staff and later entered in Excel templates for analysis. Outcomes related to distribution focused on the reach of the packs among the eligible population and the fidelity of implementation, measured as the number of complete packs as compared to partial packs distributed (Table 3). Distribution metrics for SBCC materials were not tracked explicitly, and no metrics on SBCC engagement were collected.

**Table 3:** Implementation outcome endpoints with description of data sources

Domain	Numerator	Data source for numerator	Denominator	Data source for denominator	Related survey question
Reach	# eligible individuals who received BITE pack at each distribution point	Distribution forms from implementers at each distribution point	# eligible individuals in target villages at each distribution point	Census listing by BITE staff & by implementor tally at each distribution point	N/A
Fidelity	# complete BITE packs as intended at each distribution point	Distribution forms from implementers at each distribution point	# BITE packs delivered (complete or partial) at each distribution point	Distribution forms from implementers at each distribution point	N/A
	# respondents who reported receiving complete BITE packs as intended at each distribution point	Survey at T1 and T2	# respondents who reported receiving complete or partial BITE packs	Survey at T1 and T2	In the last 60 days, or since you last saw the study team, did you receive a (COMPONENT OF PACK)?
	# respondents who reported losing pack products	Survey at T1 and T2	# respondents who reported receiving the complete or partial packs	Survey at T1 and T2	Did you lose any of the forest pack products over the last 60 days (or since you last met with the study team)? If yes, which [products]?
Acceptability (by Forest Pack tool)	# respondents who reported satisfaction with the tool	Survey at T1 and T2	# respondents who reported receiving the tool	Survey at T1 and T2	How likely would you be to recommend the TOOL to a family member or friend?
	Qualitative data	Ethnographic study	Qualitative data	Ethnographic study	N/A
Appropriateness (by Forest Pack tool)	# respondents who reported suitability of the tool based on their lifestyle/ behaviors	Survey at T1 and T2	# respondents	Survey at T1 and T2	How useful do you feel the TOOL is in your day-to-day life?
	Qualitative data	Ethnographic study	Qualitative data	Ethnographic study	N/A
Coverage (by pack)	# respondents who reported receiving the BITE pack	Survey at T1 and T2	# respondents	Survey at T1 and T2	In the last 60 days, or since you last saw the study team, did you receive a (COMPONENT OF PACK)?
Coverage (by Forest Pack tool)	# respondents who reported receiving the tool	Survey at T1 and T2	# respondents	Survey at T1 and T2	In the last 60 days, or since you last saw the study team, did you receive a (COMPONENT OF PACK)?
	# respondents who reported using the tool yesterday or last night	Survey at T1 and T2	# respondents who reported receiving the tool	Survey at T1 and T2	Did you use the TOOL yesterday or last night?
Use (by Forest Pack tool)	# respondents who reported using the tool at least once in the last week	Survey at T1 and T2	# respondents who reported receiving the tool	Survey at T1 and T2	Have you used the forest pack TOOL in the last week? If yes, how often?

#### Survey questionnaires

At T0, participants enrolled in the study, provided blood samples for dried blood spots (DBS), and completed survey questionnaires that focused on demographic and risk factor information. T0 data are reported elsewhere [20]. At T1 and T2, participants continued to provide blood samples and complete surveys that were different from that administered at T0, and which focused more on participants’ use and impressions of each of the forest pack tools. Survey questions at T1 and T2 assessed the following parameters related to the forest pack tools (Table 3):

1. Coverage: whether participants reported receiving the tool
2. Acceptability: whether participants reported to find the tool agreeable or satisfactory
3. Appropriateness: whether participants reported to find the tool to be appropriate for their lifestyle, regardless of whether they found the tool to be acceptable
4. Use: whether participants reported using the tool the previous day/night, and whether they reported using the tool in the last 60 days
5. Safety: whether participants reported having any adverse reaction which they felt were attributable to the tool

The survey was piloted with a sample of male and female Khmer and Bunong speakers to ensure questions were clear and well understood. The survey was programmed into Open Data Kit (ODK) and data were entered through the open-source platform ONA (www.ona.io), with data collected in the field using tablets. Surveys collected information on Forest Pack bite prevention products, but not SBCC materials.

### Interviews and focus group discussions

During T2, a random sample of individuals from both study sites were selected to participate in either key informant interviews or focus group discussions (FGDs) (Appendix 2). Question guides focused on respondents’ perceptions of malaria risk, their use of existing vector control tools, and their use, impressions, and attitudes toward the newly introduced forest pack tools.

Similarly, a purposeful selection of stakeholders representing both distribution partners and various health staff associated with the study (health facility staff, public health department staff, etc.) were interviewed. For these individuals, interviews focused on the perceived appropriateness of the forest pack tools for the recipient population, and the feasibility of large-scale distribution of the tools (Appendix 3).

As with the survey, interviews and FGDs were not designed to collect information on SBCC materials. However, as these were open-ended discussions with end users and stakeholders, some participants did provide their thoughts and feedback on SBCC materials during interviews and FGDs. Interviews and FDGs were audio recorded and transcribed in Khmer, and transcripts were used for data synthesis and analysis.

### Analytical frameworks and statistical analysis

Two implementation science frameworks were used to inform the design and analysis of this study. The RE-AIM framework was used for survey design and analysis, which provides a template for evaluating various metrics related to public health interventions, including: reach, efficacy, adoption, implementation, and maintenance [21–23]. The COM-B framework (Capability, Opportunity, Motivation, and Behaviour) was used to design both semi-structured question guides and analyse qualitative data from key informant interviews and FGDs elaborated into a ten domain Theoretical Domain Framework (TDF) to identify factors which promoted or hindered the use the forest pack, both during the study and in the future [24, 25]. Where possible and appropriate, data was analysed by province, gender, and target group. Quantitative survey data was analysed using descriptive statistics, primarily proportions, using Stata version 14.

## Results

### Study population

Results from the T0 survey that include a description of the population at risk, risk factors, treatment seeking behaviors, and socioeconomic factors have been previously reported [20]. In summary, 2,111 individuals were enrolled in the study at T0, with a further 824 enrolled at T1, for a total sample size of 2935 (1510 from Sen Monorom and 1425 from Phnom Srouch) across 1,303 households and 18 villages. Of the enrolled population, 37% were forest goers, 61% were forest dwellers, and 2% were forest rangers. Proportion of males (49%) and females (51%) were approximately equal overall, though the vast majority of forest rangers were male. The largest proportion of the study population was between 26–45 years (43%) and 35% were younger than 26 years. Nearly half (47%) of all participants reported farmer as their primary occupation, with the rest categorized as day labourer, forest collector/forager, and other forest-based occupations.

### Forest pack distribution

#### Reach

Forest packs were delivered to 19 villages and three ranger stations in Sen Monorom and 19 villages in Phnom Srouch simultaneously by village malaria workers with oversight by Malaria Consortium and CNM, respectively, along with the two provincial health departments. Based on the most recent estimates of village populations, and the estimated proportion of forest exposed individuals within those villages, 85% of the target population received forest packs in Sen Monorom (Reach), and between 72–102% in Phnom Srouch, depending on the distribution timepoint (D1–D4) (Table 4). The 102% reach in Phnom Srouch reflects imprecise village population numbers and population-at-risk estimates.

**Table 4:** Reach and fidelity, calculated using distribution data, by operational district

	Sen Monorom			Phnom Srouch		
	Complete Pack as intended	Partial Pack (missing > 1 product)	Complete + Partial Pack	Complete Pack	Partial Pack (missing > 1 product)	Complete + Partial Pack
D1	48%	37%	85%	–	72%	–
Num	1979	1521	3500	–	2226	–
Den	4137	4137	4137	3079	3079	3079
D2	84%	0%	85%	102%	0%	1.02
Num	3493	3	3496	2242	0	–
Den	4137	4137	4137	2194	2194	2194
D3	35%	49%	85%	–	73%	–
Num	1457	2047	3504	–	2263	–
Den	4137	4137	4137	3079	3079	3079
D4*	85%	0%	85%	–	–	–
Num	3500	0	3500	–	–	–
Den	4137	4137	4137	–	–	–

Num: Numerator = # eligible individuals who receive BITE pack at each distribution point; Den: Denominator = # eligible individuals in target villages at each distribution point

^*^Distribution data is not available for Distribution 4 in Phnom Srouch as no forest packs were delivered during this round

#### Fidelity

Complete forest packs (containing BiteBarrier, topical repellent, and SBCC materials alongside etofenprox solution used by VMWs to treat clothing) were intended to be distributed at D1 and D3, while partial packs (i.e., without etofenprox solution) were intended to be distributed at D2 and D4. The distribution data presented in Table 5 indicates that in both provinces, the initial distribution saw fewer complete packs delivered than planned for D1 and D3 given limited implementation of etofenprox treatment (Fidelity). Nearly all individuals targeted received a partial forest pack as intended (etofenprox solution not included) at D2 and D4. In Phnom Srouch, distribution data is incomplete, so it is not possible to calculate the fidelity of the distribution.

**Table 5:** Fidelity and coverage of the forest packs among target populations in Sen Monorom and Phnom Srouch, Cambodia, reported by users through cross-sectional surveys

	Sen Monorom		Phnom Srouch	
	T1	T2	T1	T2
Fidelity–complete	18%	40%	1%	4%
Num	185	431	8	39
Den	1052	1073	1011	945
Coverage (Complete Pack)	17%	40%	1%	4%
Num	185	431	8	39
Den	1113	1089	1079	958
Coverage (BB)	93%	98%	91%	98%
Num	1036	1068	979	940
Den	1113	1089	1079	958
Coverage (TR)	93%	98%	93%	98%
Num	1036	1069	1007	940
Den	1113	1089	1079	958
Coverage (ETO)	17%	40%	1%	5%
Num	191	434	10	44
Den	1113	1089	1079	958

Coverage Num: Numerator = # eligible individuals who receive BITE pack at each distribution point; Coverage Den: Denominator = # eligible individuals in target villages at each distribution point

Because VPSR and TR were reliably included in nearly all distributions, partial packs in this study generally refer to packs missing ETO at D1 or D3.

### Survey results

At T1, most recipients reported receiving only a partial pack, particularly in Phnom Srouch, primarily due to limited coverage of etofenprox clothing treatment (Table 5). By T2, fidelity of the pack had improved in both sites, though coverage remained higher in Sen Monorom. Reported coverage of BiteBarrier and topical repellent were consistently high (> 90%) between the two surveys and two sites. No meaningful differences in fidelity or tool coverage were observed by target group or gender.

Overall, user-reported acceptability and perceived appropriateness of all three tools were high among those who received the tools and increased from T1 to T2 as participants became more familiar with their use (Table 6). By T2, nearly all users described the tools as acceptable and suitable for their lifestyle. Reported use—both “yesterday/last night” and “within the last week”—was also high across most tools, with some variation by target group. Forest dwellers consistently reported the highest use, especially for BiteBarrier and treated clothing, while forest rangers reported slightly lower usage. Topical repellent was used less frequently overall, especially at T1. Usage patterns were broadly similar across genders.

**Table 6:** Fidelity, acceptability, appropriateness, and use of forest packs reported by end users in cross-sectional surveys in Sen Monorom and Phnom Srouch, Cambodia

	Goers		Dwellers		Rangers	
	T1	T2	T1	T2	T1	T2
Metric	%					
Fidelity–complete packs	7	26	10	21	22	30
Acceptability (BB)	81	93	72	95	80	96
Acceptability (TR)	83	93	71	92	78	93
Acceptability (ETO)	92	94	85	96	78	100
Appropriateness (BB)	85	97	83	97	80	90
Appropriateness (TR)	85	96	81	95	90	86
Appropriateness (ETO)	96	97	99	99	67	100
Use (BB)—Last Night	83	89	84	93	70	78
Use (BB)—In Last Week	85	95	87	97	85	88
Use (TR)—Last Night	69	72	56	70	45	51
Use (TR)—In Last Week	83	91	79	90	90	78
Use (ETO)—Last Night	45	74	59	76	44	93
Use (ETO)—In Last Week	86	76	88	98	67	50

### Self-reported safety of BITE tools

Among respondents who reported receiving the tools, adverse events were uncommon, with only 3.3% of participants reporting any side effect and a total event rate of 3.9% across both surveys (Table 7). The most frequently reported symptoms were headache, difficulty breathing, and responses categorized as “other.” BiteBarrier had the lowest rate of reported side effects (1.7%), while topical repellent had the highest (4.9%). Headache was the most frequently cited symptom across tools, except for treated clothing, where “other” side effects were most common.

**Table 7:** Self-reported side effects perceived as attributable to each intervention

	BiteBarrier		Topical		ETO	
	T1	T2	T1	T2	T1	T2
n	2015	2008	2043	2009	201	478
Rash on chest	1	1	0	2	2	0
Rash on back	0	0	2	2	2	0
Rash on legs	0	0	6	2	1	0
Rash on arms	4	3	11	12	2	0
Irritation or burning in eyes	0	0	1	0	1	0
Irritation or burning in nose	0	0	2	0	0	0
Irritation or burning in mouth	1	0	1	2	0	0
Shortness of breath	0	0	1	5	0	0
Difficulty Breathing	1	4	12	21	0	1
Coughing	1	2	1	2	0	0
Productive cough	1	1	0	0	0	0
Allergic reaction	2	9	3	7	2	2
Headache	13	7	60	24	1	2
Other	19	0	54	14	8	1
Any adverse event	42	25	125	75	15	6
Total	43	27	154	93	19	6
Total %	2.1%	1.4%	7.8%	4.6%	9.5%	1.3%

Very few participants sought medical attention due to side effects. Across all tools and time points, fewer than 1% reported visiting a health facility, and only 1.5% reported needing any treatment. No participants reported seeking care in response to wearing treated clothing. One individual reported an overnight hospital stay, perceived to be related to topical repellent use.

### Interview and focus group discussion results with end users

In Sen Monorom, interviews were held with 27 individual end users (22 males, 5 females), and four focus groups were held with 28 end users (14 males, 14 females) across four villages (one focus group discussion in each village). In Phnom Srouch, 29 interviews were conducted (22 males, 7 females), and 30 end users (13 males, 14 females) participated in four focus group discussions (one in each village). In Sen Monorom, 56% of all interviews were with forest goers, 29% with forest dwellers, and 15% with forest rangers. In Phnom Srouch, 70% of all interviews were with forest goers, and 30% were with forest dwellers. All interviews and focus group discussions were conducted during timepoint T2, when people had access to the forest pack tools for approximately four months.

Using the COM-B framework, study investigators explored the barriers and facilitators of use for each of the tools to provide context to the survey data.

### Facilitators of use

Participants highlighted ease of use and adaptability as key facilitators for product use. Tools were used in combination and individually, suited to different settings and activities—for example, repellent and treated clothing while traveling or working, and BiteBarrier at home.

“We can use it [topical repellent] anytime and anywhere. Sometimes, when I go to the toilet at night, I take it with me to spray [themselves]. So I can take it with me everywhere I go.”** Key Informant Interview, Sen Monorom, Female Age 26**

“There is nothing difficult with the treated clothing. After treatment, we can wear it and don't have to carry it in a bag. We wear it like our regular clothes to the forest. This is relatively easy. We can even sleep with the clothes on in the forest.” **Key Informant Interview, Sen Monorom, Male, Age 32**

Another facilitator of use was that the tools were provided to participants by trusted members of their community. These individuals were mainly village malaria workers (VMWs) but also included village chiefs and local teachers. Having these trusted community members provide the forest packs to participants, explain their uses, and facilitate discussions about the new tools helped participants to trust in the tools.

“The Phet Smackchet [VMW] told us how to spray the product on our arms and legs. He said there was no problem. So, I followed his advice. I trusted the phet. Otherwise, I would not dare to use it. Other people see that the product is effective and want to have it.” **Focus Group, Phnom Srouch, Male, Age 45**

In addition, women in the community are described as playing a crucial role in facilitating use of mosquito bite prevention products by husbands and sons going to the forest.

“My wife always prepared my bag to take to the forest. So, my wife constantly reminds me to use the spray [topical repellent]. How could I forget when she had already put it in my bag? The VMW also encourages me to use the product to protect myself from mosquitoes. But it is my wife that reminded me not to forget.”** Key Informant Interview, Sen Monorom, Male, Age 37**

### Barriers to use

Barriers included confusion around how to use the tools safely and appropriately, with misuse of tools reported by some participants:

"I used it [topical repellent] at home. When it is getting dark, I sprayed topical repellent around the bed, in a dark place in the corner of the house. I sprayed on clothes in the closet. In general, when the sky getting dark, the mosquitos come out, so I just spray to protect ourselves."** Focus Group, Sen Monorom**

Some participants misunderstood written instructions included in printed SBCC materials.

“I did not understand the instruction; I thought it was about the product's formula.”** Key Informant Interview, Phnom Srouch, Male, Age 48**

BiteBarrier was seen as impractical in outdoor conditions due to wind, rain, and movement.

“Taking the [VPSR] to the forest is problematic because it is like a sheet of paper; the rain can quickly destroy it.” **Focus Group, Sen Monorom**

Washing treated clothing in streams raised concerns about water contamination.

“The challenge with treated clothing is that we have to wash them after being treated, and we cannot use the water in the stream or pond. The forest goers who went to the forest for an extended period had to wash their clothes some time, and they did not bring a bucket or something to wash them with. So, they washed directly inside the stream or pond where they stayed. Then they cannot use the water.” **Key Informant Interview, Phnom Srouch, Male, Age 40**

Access to clothing treatment or re-treatment was also a challenge.

“The treated clothing is the most complicated because our staff has to come to the regional [forest ranger] station to get their clothes treated, and they only come once a month. Moreover, after being treated, they have to wash them, and this makes it difficult for them because we don’t have a place, and they cannot wait to get their clothes cleaned and take them back to their station.” **Key Informant Interview, Sen Monorom, Male, Age 36**

### Interviews with implementers

The study team conducted 20 in-depth key informant interviews with government (n = 18) and NGO (n = 2) implementers, including 11 interviews in Phnom Srouch (10 males, 1 female) and nine in Sen Monorom (6 males, 3 females). Government respondents included VMWs, heads of health centers, and provincial and national health officers, including from CNM. In-depth interviews focused on the feasibility and sustainability of a large-scale delivery of the BITE forest packs, with consideration of each tool independently. In this case, feasibility relates to the logistics, distribution method, and frequency, and sustainability relates to the ability to maintain distribution over a large area and time scale.

### Appropriateness of tools

Implementers widely viewed the BITE forest pack as appropriate for Cambodia’s malaria elimination context.

"The BITE Forest Package is very valuable for our community because we are at risk for malaria. Our village is adjacent to the forest and far from the health center. The villagers, particularly men, go to the forest, and most older people go to the farm in the forest. And with these products available, I think they are very useful and beneficial for my village."** Sen Monorom, Village Malaria Worker**

However, many suggested improvements to distribution timing and methods.

"If the project will continue the distribution, it is better to do it in the rainy season, and people would use more. The product was distributed during the dry and windy, so not many people use them."** Sen Monorom, Village Malaria Worker**

Views on distribution frequency were mixed. Some supported the current schedule; others preferred a more flexible approach based on forest exposure. But increasing frequency raised concerns about the burden on VMWs.

"As I observed, the frequency of delivery that we are applying now is appropriate when with the period of distribution and the proportion of the people going into the forest. Normally, the forest goers do not go to the forest daily; they only use the product when they go there."** Phnom Penh, CNM Staff**

"If they [forest goers] are not going to the forest frequently or only go once in a while, then they would not use up the product, which will have left over and eventually it cannot be used anymore. Giving the product to people every month can be wasteful, especially for those that did not go to the forest frequently. So, we have to target those who go to the forest often, and knowing how long they stay there, we can give the product accordingly."** Phnom Srouch, Village Malaria Worker**

Distribution was meant to occur at central points but often shifted to house-to-house delivery to ensure full coverage.

"I know the individuals at risk for malaria, so I go to their houses to give the products. If I asked them to come to my house to take the product, they would not come. So I have to go directly to their houses."** Sen Monorom, Village Malaria Worker**

### Feasibility of continued distribution and use

Respondents highlighted logistical challenges in reaching remote villages and distributing packs on schedule. Many still expressed strong personal motivation to support the effort.

"Yes, I am exhausted sometimes, but I also have to spend on fuel going from house to house. Many people are in my village, and my wife helped me with the distribution. Sometimes, I was able to gather 20 to 30 people to come to collect the products, so my wife had to help me, and I did the recording of names. I want to do this because there are a lot of benefits for society and the family. We have to help each other and care for each other."** Phnom Srouch, Village Malaria Worker**

Implementers supported expanding BITE tool delivery to broader geographic areas and high-risk groups, including forest campers, migrants, and remote communities at risk of malaria and dengue.

"When we think about expansion, we have to be specific about the location, the endemic area of malaria. We should only expand in some places. For example, with our previous experience with the distribution of treated mosquito nets to Pai Lin and Banteay Meanchey provinces, in the first year, we distributed them to many areas. Still, the following year stopped because there was no more malaria in these two provinces. Except in Pai Lin, we continued the distribution to some pockets of areas that still have malaria. Therefore, it is the same with our BITE products; we have to consider this issue, expanding to specific areas with risks.

Moreover, we should think about more than just malaria. It is essential to think about dengue fever as well to have a broader coverage. Therefore, the BITE products can protect both from malaria and dengue and select the right target areas."** Sen Monorom, CNM Staff**

However, concerns were raised about scaling up—particularly around staffing capacity and funding limitations.

"If we expand to all villages to cover all the population, we will face challenges regarding human resources, especially for the health center staff and the VMWs. Firstly, it will be challenging for them to do the follow-up after the distribution of the products. We need to follow up with users so that we know they use the products and use them correctly. So, to cover all villages and everyone in the village, there will be a lot of work for VMWs, especially the VMWs who are the chief or deputy chief of the village; they would only have time to do some of the work. Therefore, it falls to the health center staff that must engage in follow-up."** Phnom Srouch, Health Centre Staff**

"I think the main challenge in expanding the distribution and delivery of BITE products in this area is the difficulty with the mobile migrant population, especially related to data management. If we inform the people that we provide the products to everyone going to the forest, the mobile migrants would all come to get the products, but we would be unable to manage them."** Phnom Srouch, Provincial Malaria Supervisor**

### Sustainability of forest pack implementation

Respondents emphasized the need for more training and follow-up to support proper tool use and long-term uptake.

“It is important to have follow-up activities after we distribute the products to the people in the community to see how people use the products. We need to know if people have problems with the product or if they use the product correctly so that we can inform them further."** Sen Monorom, Health Centre Staff**

"I think there is sustainability with these products because most people using them live in the forest and make their living from the forest. So, if we provide the products, they would use them. How often and in what way do we want to distribute the products to the people so they can use them regularly? The current distribution method can be sustainable because we use local resources such as the VMWs and the village chiefs."** Sen Monorom, Health Centre Staff**

Many noted the tools’ relevance beyond malaria, highlighting continued use to prevent nuisance mosquito bites.

"BITE Forest Package currently is very popular for people that go to the forest. Even though in the future we eliminate malaria, mosquitos will always exist; therefore, the BITE forest Package will be relevant in protecting against mosquito bites, and people will continue to use it. Even without disease transmission, a mosquito bite can disturb their sleep and work, so people will continue using the products.”** Phnom Penh, CNM Staff**

## Discussion

### Summary of implementation

This study evaluated the acceptability and feasibility of distributing forest packs containing novel bite prevention tools to approximately 5744 individuals at higher risk for malaria over a four-month period in *P. falciparum* epidemiologic hotspots in two operational districts in Cambodia. During the period of this study—October 2022 to February 2023—there were a total of 198 malaria cases in Sen Monorom (48 *P. falciparum,* 150 *P. vivax*) and 120 malaria cases in Phnom Srouch (2 *P. falciparum*, 114 *P. vivax*, 2 *P. malariae*, 2 mixed) (data from Cambodia National Malaria Information System (MIS)).

### Acceptability and appropriateness of the forest pack tools

Study results indicate that, overall, the forest pack bite prevention tools were well-accepted by end users and implementers and were perceived to be safe. After approximately four months of experience with the tools, acceptability of all forest pack tools was reported to be greater than 90% in both districts and across all target groups, and the tools were considered appropriate for their living and working contexts. Reported use of the bite prevention tools was heterogeneous: at the T2 survey, 50% of forest rangers versus 98% of forest dwellers reported using treated clothing in the past week among those for whom their clothes were treated with the etofenprox solution. The VPSR was the most frequently used tool, with reported use in the last week ranging from 88% for forest rangers to 97% for dwellers. Tool use did not vary significantly by age or gender.

Participants reported that the VPSR was best suited for village settings, particularly inside and around structures. In contrast, treated clothing was reported to be most useful when mobile in forests and crop fields. Topical repellent was described to be useful in both settings, with users indicating frequent use in both forested settings and within villages, especially when cooking or socializing outside of the home. During key informant interviews with project implementors, both government and NGO staff reflected that they believed that each of the forest pack tools were appropriate bite prevention products for forest-exposed and mobile populations, though perspectives varied on which tool was most preferred, and in which context. Both end-users and implementors appreciated that the combination of tools—especially when paired with ITNs and ITHNs—had the potential to offer 24 h protection from mosquito bites [7].

### Differential use patterns among target groups

An interesting pattern emerged with regard to etofenprox-treated clothing: forest rangers reported higher use than other groups. While this study was not designed to fully investigate the reasons for this difference, previous field experience and qualitative findings offer some potential explanations. Rangers spend a significant portion of their time in forested areas, which may increase their awareness of mosquito exposure and the value of protection. Additionally, rangers typically wear standardized uniforms, which were treated with etofenprox during the intervention. This may have made application easier, reduced variability in treated garments, and encouraged more consistent use. These contextual and occupational factors may help explain the higher uptake of treated clothing in this group, and suggest that uniform-based interventions could be a promising strategy in other professional or semi-professional populations operating in high-risk environments.

### Community leadership and gender roles as catalysts for uptake

Important facilitators of tool use included engaging SBCC and SBCC delivery from trusted messengers, including village and health representatives and women within households. End users frequently indicated the influence of village leaders and VMWs on their use and acceptability of the tools. Delivering tools through VMWs was perceived as positively contributing to tool uptake, since VMWs are known in the communities, speak local languages, and understand the communities in which they work, including timing and frequency of population mobility. The role and influence of women in the communities was also frequently reported through interviews and focus group discussions. Wives and mothers described reminding their husbands and sons to bring the forest pack tools with them while travelling or working in the forest.

### Logistical realities of delivering forest packs

Implementers noted difficulties in delivering the forest pack tools to some communities due to the combination of heavy, bulky products and poor road infrastructure, especially during the rainy season. Implementers related that, while they felt the forest pack was appropriate for the target populations, getting the packs to those populations could be challenging and may compromise the sustainability of the forest pack as a long-term intervention.

The treatment of clothing with etofenprox-based solution proved to be the most challenging intervention to deliver to target populations. Initially, implementors planned to have all eligible individuals in a village come to a central location, bringing with them their clothing for treatment, and staff would apply the etofenprox solution to clothing in bulk. There were several factors which made this methodology impractical. First, not all people are available at the same time. Second, not all people were comfortable having their clothing treated in public; some end users indicated that, because their clothing may be dirty or tattered, they were embarrassed to bring them to be treated along with others’ clothing. Third and finally, people wear and wash clothing with different frequencies. This etofenprox solution can last up to 25 washes on treated clothing, and since individuals wear different articles of clothing for different lengths of time, under different conditions, and wash them on different schedules, a bulk-treatment scheme may lead to inadequate, undertreatment of some clothing and wasteful, over-treatment of other clothing [8].

For these reasons, implementors switched from communal, bulk-treatment of clothing to household-based treatments. VMWs and others assisting with forest pack delivery communicated with individual households to determine when and where treatment of clothing was appropriate for their family, which led to higher uptake and better understanding of the treatment. However, this household-based distribution of etofenprox treatment was much more time consuming and laborious for VMWs and other staff. Furthermore, while staff had been provided with large (7.5 Litre) pump sprayers, adequate for treating a large amount of clothing at once, teams ultimately preferred smaller (2 Litre) handheld pumps for treating clothing at the household level, as they are easier to transport and manage. These challenges resulted in low reach and coverage of etofenprox treated clothing and low fidelity of the intervention at the first and third distribution time points that was intended to include etofenprox treatment of clothing as part of the complete pack.

### Implementation fidelity and communication

Implementation varied by OD. Low fidelity primarily reflected incomplete delivery of ETO treatment at D1 and D3, rather than absence of VPSR or TR. In Phnom Srouch, there was no D4 distribution and incomplete distribution data for D1-D3. NGO oversight and support in Sen Monorom, as well as frequent communication and problem-solving between the NGO, VMWs, clinic staff, provincial and district health teams, and the research team, likely enabled higher-quality data and distribution. As a result, reach, fidelity, and coverage of the intervention was higher in Sen Monorom. These outcomes suggest the importance of effective communication, supervision, oversight, and adaptive implementation, especially when new products are introduced.

Evidence-based and sustained SBCC has been shown to significantly improve uptake of bite prevention tools [26, 27]. Consistent with this best practice, the project team developed evidence-based SBCC materials based on insights from an initial formative assessment conducted among similar high-risk populations in Sen Monorom [10]. SBCC formats were selected based on project timeline and budget parameters. Consistent with formative insights, SBCC focused on explaining and motivating correct tool use. Respondents and implementers alike reinforced the challenge of text-based materials and suggested earlier use of more engaging SBCC formats, such as the animation video.

### Timing of distribution and exposure

When participants reported receiving the products and understood how to use them properly and safely, there was high acceptability and use. However, the timing of distribution (October–January) did not fully align the rainy season (typically August–December). End users suggested that better alignment with the rainy season would likely increase the frequency and consistency of tool use. These findings suggest that targeting periods of higher mosquito density is essential for optimizing protection.

Although the project team had initially planned for earlier distribution, delays in product procurement, ethical approvals, and preparatory activities pushed implementation into the dry season. A longer planning and preparation window could have enabled earlier tool distribution, more in-depth community engagement, and more robust training and SBCC efforts.

### Innovation and future applications

The relatively short, four-month implementation window limited sustained tool use and longer-term observations. A longer exposure period may have provided insights into seasonal tool preferences and allowed participants to adjust their habits to align with mosquito biting trends and disease risk over time.

Future innovations may help overcome some of the logistical and usage challenges reported. For example, efforts to extend the efficacy of VPSRs to a year or more would reduce distribution frequency and increase convenience. Likewise, factory-treated clothing could offer a more durable and user-friendly solution than community-based spraying. However, challenges such as cost, sizing, and cultural preferences must be considered, especially when designing interventions for diverse user groups [28]. These tools may also be applicable in humanitarian settings and for other vector-borne diseases, including Aedes-borne viruses, for which evidence of efficacy is emerging [7, 29, 30].

### Study limitations

This study was not designed to evaluate the epidemiological effectiveness of the forest pack tools against malaria transmission. The extremely low incidence of *P. falciparum* malaria in Cambodia made conducting efficacy or effectiveness trials infeasible. Although semi-field and field studies have demonstrated entomological efficacy of individual and combined tools [7, 8], their combined epidemiological impact remains to be confirmed. Additionally, tool use may vary by context, timing, and vector exposure, making it difficult to generalize the findings beyond the study population.

Another limitation is the lack of systematic data on how SBCC materials were distributed, observed, or used by end users. While qualitative interviews and focus groups provided insight into their perceived value, the study did not formally track the extent of SBCC material coverage or measure whether materials were actively consulted in daily practice. As a result, the ability of the study team to assess the contribution of SBCC to the overall implementation outcomes is limited. The study targeted specific forest-exposed populations but did not include all high-risk groups. Notably, highly mobile or migrant populations, who are often reservoirs for malaria transmission, were excluded due to practical limitations on follow-up and repeated data collection [31].

Lastly, while the study relied on multiple data sources—quantitative surveys, focus groups, and key informant interviews—there are inherent limitations to self-reported data. One of these limitations is the potential for social courtesy bias in participant responses. Although data collection was conducted by independent research staff who were not part of the VMW network or government health system, some participants may have still perceived them as connected to the intervention or as authority figures. This perception could have led to participants offering more favorable responses regarding forest pack tools, their use, or satisfaction, regardless of their true experience. It is also possible that social courtesy bias varied by participant group; for instance, forest rangers—due to their professional status and experience working with health or government stakeholders—may have felt less compelled to give overly favorable responses than forest goers or dwellers, though this was not formally assessed. While efforts were made to ensure impartiality, build rapport, and emphasize that honest feedback was valued and without consequence, the potential for social desirability bias remains. Future studies could further mitigate this risk by incorporating more anonymous or indirect response methods to validate self-reported data.

## Conclusion

This is the first study to evaluate large-scale implementation of new bite prevention tools combined in a forest pack to high-risk populations for malaria. The forest pack was found to be acceptable and appropriate for preventing mosquito bites among highly exposed populations in two operational districts in Cambodia. Future distribution of forest packs containing novel bite prevention tools should prioritize early planning, early SBCC implementation, and learning-by-doing approaches to delivering mosquito bite prevention tools to avoid some of the challenges faced in this study. Further epidemiological studies are needed in higher-burden settings to demonstrate the impact of combined bite prevention tools in a pack on malaria infection among high-risk populations using tools in open structures and outdoors when working, and transmission modeling can provide insights when epidemiolocal studies are not feasible. New tools and approaches will continue to be critical for countries like Cambodia pursuing malaria elimination in the near-term and for populations at-risk across malaria endemic settings where existing interventions are insufficient to prevent vector biting.

## Supplementary Information

Additional file1 (PDF 774 KB)

Additional file2 (PDF 1417 KB)

Additional file3 (PDF 74 KB)

Additional file4 (PDF 72 KB)

Additional file5 (PDF 5485 KB)

Additional file6 (PDF 5474 KB)

Additional file7 (PDF 426 KB)

Additional file8 (PDF 128 KB)

Additional file9 (PDF 174 KB)

Additional file10 (PDF 154 KB)

## Data Availability

All data generated or analysed during this study are included in this published article and are available upon request.

## Abbreviations

BB: BiteBarrier
BITE: Bite interruption toward elimination
CNM: Cambodia’s National Center for Parasitology, Entomology, and Malaria Control
COM-B: Capability, Opportunity, Motivation, and Behaviour
FGD: Focus group discussion
GMS: Greater Mekong subregion
ITC: Insecticide treated clothing
KII: Key informant interview
LLIHN: Long-lasting insecticide treated hammock net
LLIN: Long-lasting insecticide treated net
NGO: Non-governmental organization
OD: Operational district
RE-AIM: Reach, Efficacy, Adoption, Implementation, Motivation
SBCC: Social and behavioural change communication
TDF: Theoretical domain framework
TR: Topical repellent
VMW: Village malaria worker
VPSR: Volatile pyrethroid spatial repellent
