# Supplementary material for: Evaluating the acceptability and feasibility of new mosquito bite prevention tools in a “forest pack” to support malaria elimination in Cambodia
Source: Malar J. 2025 Nov 27;24:443. doi: 10.1186/s12936-025-05682-2 (PMC12715958; doi:10.1186/s12936-025-05682-2)
Supplement: Supplementary file 1 — Additional file1 (PDF 774 KB) [file 12936_2025_5682_MOESM1_ESM.pdf]

How to use three mosquito bite prevention tools

Take all mosquito bite prevention tools with you when you go to the forest. Wear your treated clothing, apply your topical repellent, and take your PIRK to hang near you while you are resting or sleeping.

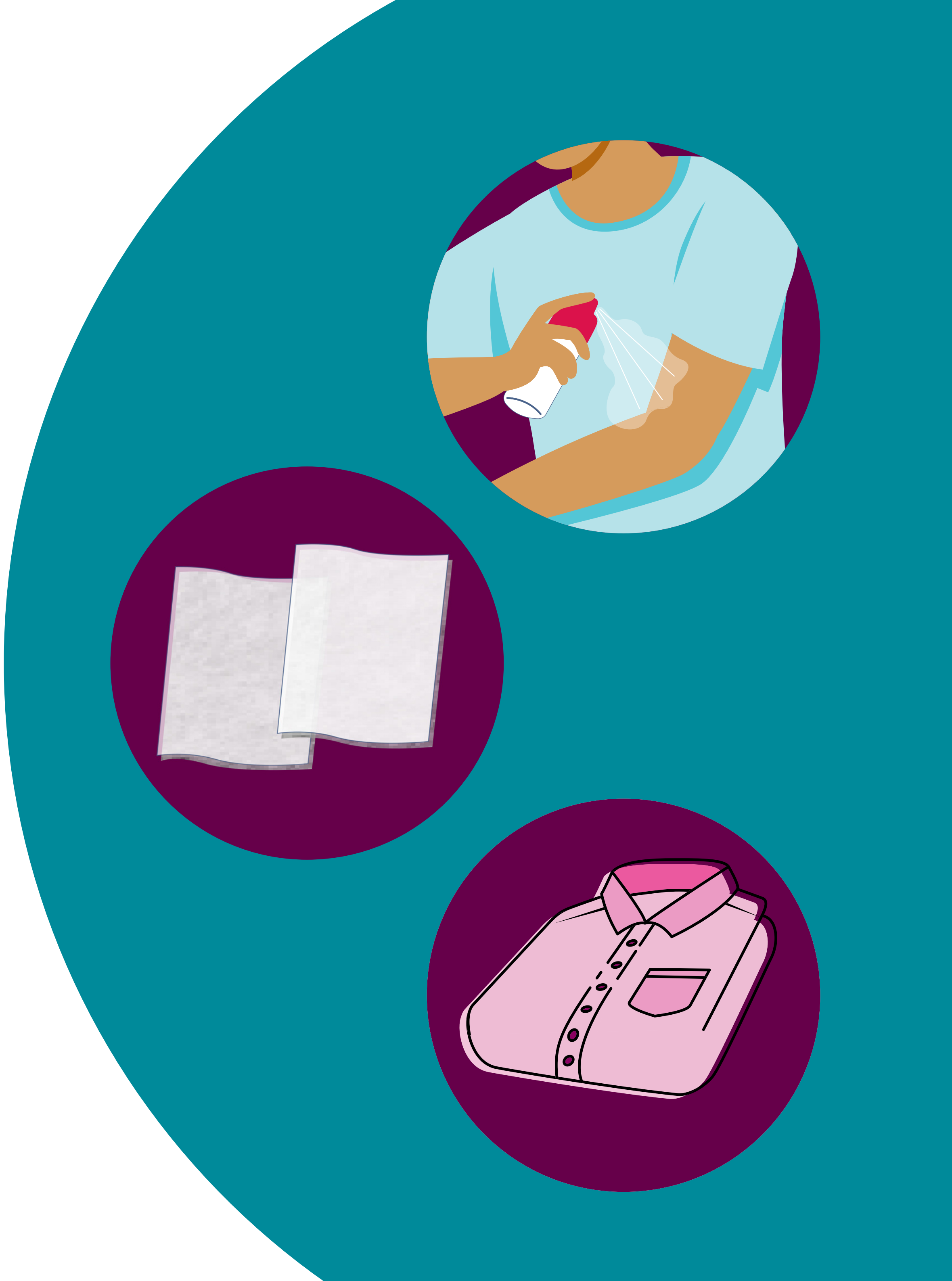

Malaria Elimination Initiative

UCSF

Institute for Global Health Sciences

Spatial repellent - PIRK

What is it?

One PIRK is made of two small rectangular sheets of woven material. They look like sheets of paper

What is it for?

The PIRK creates a protective "bubble" which mosquitoes do not like to enter

How is it used?

The PIRK is hung on a wall or from the roof, just about head height

Once it is hung, it will be working. No need to move it or do anything else

How can it be used with other vector control tools?

Use the PIRK alongside any other vector control tools you use

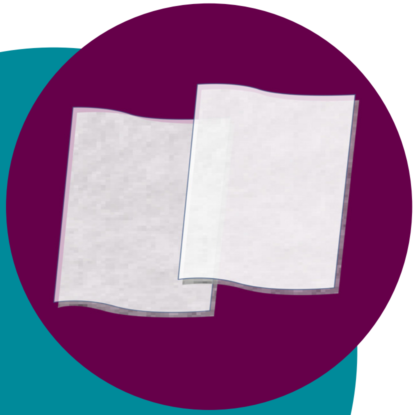

DO

- Before use, store in a cool, dry area out of sunlight, and in the plastic bag provided until ready to use
- Put on gloves before opening the package and only open when you are ready to hang and use the sheets
- Hang the sheet above your head indoors or outdoors, in your home or a structure in the farm or forest, or on a tree
- Hang the sheet in the area of your home where you spend the most time
- Sheets should be out of children's reach
- Use the sheet as much as possible, even when are also using a treated net or hammock
- Wash your hands with soap and water after hanging
- Replace sheets after 30 days, or when you receive a new set from project staff
- Take the sheet with you as you move from home to forest farm or ranger station and back again: treated sheets can be used safely in different settings

DO NOT

- Hang the sheets in an area where people do not spend time (i.e., animal structure)
- Hang the sheet where it will be exposed to direct sunlight or rain for long periods
- Use sheets if your packaging has been opened before you are ready to use the product
- Get the sheet wet. If you are bringing the sheet to the forest, keep it in a plastic bag to protect it from the rain and take it out when you are ready to set it up and use it
- Give the sheet to someone outside your family or ranger group

Safety considerations

- PIRK is odorless - no smell!
- Touching the PIRK with skin can cause rash or irritation
- Use gloves or a plastic bag when touching the PIRK
- If you develop a rash or feel irritation, visit a local health centre - symptoms should go away soon
- Store the PIRK away from where children can reach
- After handling PIRK, wash your hands with soap and water

Product specific usage

Topical repellent

What is it?

A clear liquid spritz that comes in a small plastic bottle with a finger-pump, containing either 20% Picaridin or 15% DEET

What is it for?

After spraying your skin with the body repellent, mosquitoes are repelled from landing on you and biting

How is it used?

It is sprayed on the skin and is effective for up to 8 hours

How can it be used with other vector control tools?

Use the topical repellent alongside any other vector control tools you use

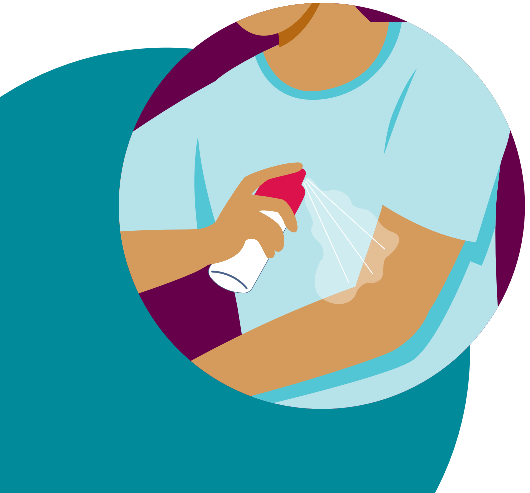

DO

- Store in a cool, dry area out of sunlight until ready to use
- Spray onto exposed skin
- Use the repellent in combination with other bite prevention tools, like treated clothing or treated sheets
- Apply the repellent up to three times per day when you are not sleeping or resting under a bed or hammock net
- Keep the repellent bottle away from children

DO NOT

- Spray the repellent into your eyes or mouth
- Give the repellent to someone outside of your family or ranger group

Safety considerations

- The topical repellent has a slightly sweet smell - the odor is harmless
- Do not get the topical repellent in your eyes or mouth
- Store the topical repellent away from where children can reach
- Only needs to be applied twice per day as it is effective for up to 8 hours

Treatment for clothing

What is it?

Treated clothing includes any piece of clothing that has been sprayed with an insecticide

What is it for?

Treated clothing is not meant to repel mosquitoes, instead it is meant to kill mosquitoes that come into contact with the clothing

How is it used?

Clothing is sprayed with the insecticide and worn the same way you wear other clothing

Treated clothing with etofenprox can remain effective for 25 washes

How can it be used with other vector control tools?

Use treated clothing alongside any other vector control tools you may use

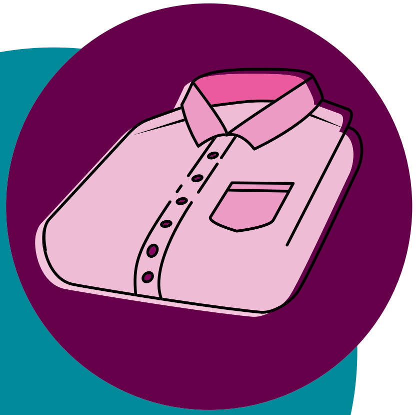

DO

- Wear treated clothing the same way you wear other clothing
- Make sure you wear treated clothing when you are in the forest, outdoors, or not resting under a treated net or hammock
- Treated clothing requires no special care, compared to other clothing
- Wash treated clothing per instruction and ensure water used to wash the clothing is poured into the ground (vs other water sources that may be used for drinking or aquatic animal habitats)
- If the newly treated clothing has a strong or unpleasant smell, rinse once to help alleviate this

DO NOT

- Let people outside of your family or ranger group use your treated clothing. They have not been trained the way you have
- Wash treated clothing in streams or rivers, or other sources that may be used for drinking or aquatic animal habitats

Safety considerations

- Treated clothing may have an odor - the odor is harmless and it will dissipate with use and washing
- You may experience a slight rash or itchiness when clothing is freshly treated - you can rinse the clothing to help reduce this
- Wash treated clothing per clothing instruction
