# Supplementary material for: Evaluating the acceptability and feasibility of new mosquito bite prevention tools in a “forest pack” to support malaria elimination in Cambodia
Source: Malar J. 2025 Nov 27;24:443. doi: 10.1186/s12936-025-05682-2 (PMC12715958; doi:10.1186/s12936-025-05682-2)
Supplement: Supplementary file 2 — Additional file2 (PDF 1417 KB) [file 12936_2025_5682_MOESM2_ESM.pdf]

របៀបប្រើឧបករណ៍ការពារ មូសខាំទាំងបីមុខ

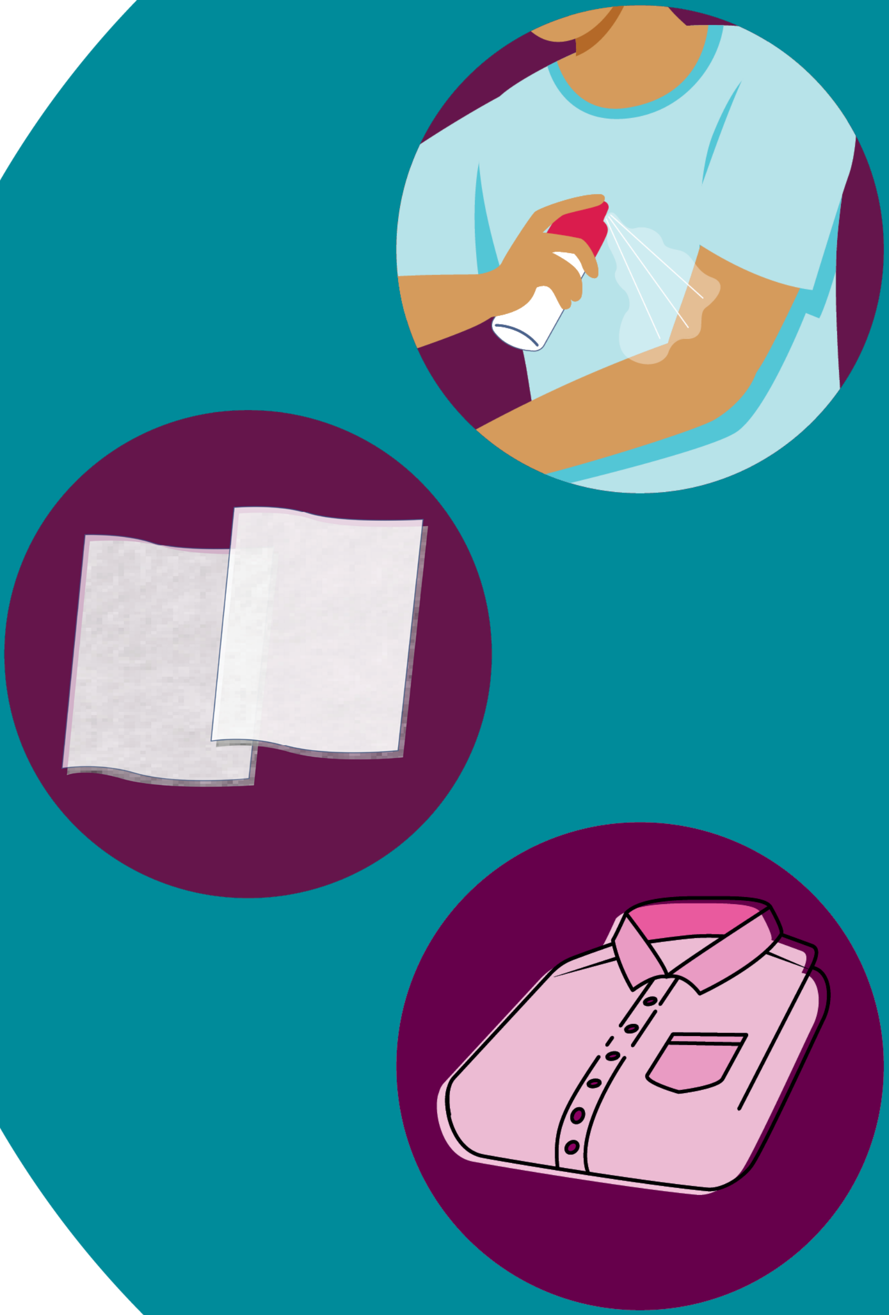

សូមយកឧបករណ៍ការពារមូសខាំទាំងអស់ទៅ ជាមួយ ពេលអ្នកទៅព្រៃ។ ស្លៀកសម្លៀកបំពាក់ ដៃជ្រលក់ថ្នាំ លាបថ្នាំការពារមូសខាំលើស្បែក របស់អ្នក ហើយយក ភើករបស់អ្នកទៅព្យួរនៅ ជិតអ្នក នៅពេលអ្នកកំពុងសម្រាក ឬគេង។

Malaria Elimination Initiative

UCSF

Institute for Global Health Sciences

Australian Government

Department of Foreign Affairs and Trade

IVCC

Building Partnerships
Creating Solutions
Saving Lives

លក្ខខណ្ឌពិសេសនៃការប្រើប្រាស់ បន្ទះការពារមូសខាំសំរាប់ព្យួរ ផើក (PIRK)

តើអ្វីទៅជា PIRK ?

មួយកញ្ចប់នៃ PIRK ត្រូវបានធ្វើជាត្បាញពីរសន្លឹកក្រដាស ចតុកោណ។ គេមើលវាដូចជាសន្លឹកក្រដា

PIRK ធ្វើឡើងដើម្បីអ្វី ?

បន្ទះ PIRK ត្រូវបានបង្កើតឡើងសម្រាប់ការការពារ “bubble” ដែលមូសមិនចូលជិតអ្នក

តើវិធីប្រើប្រាស់ PIRK យ៉ាងដូចម្ដេច ?

បន្ទះ **PIRK** ដែលគេប្រើសម្រាប់ព្យួរលើជញ្ជាំង ឬពីលើដំបូល មិនតែប៉ុណ្ណឹងព្យួរកន្លែងខ្ពស់

ការព្យួរ PIRK ម្ដងៗ វានឹងមានប្រសិទ្ធភាព (៣០ថ្ងៃ) ដែលមិនតម្រូវផ្លាស់ប្ដូរវាទេ មិនចាំបាច់ធ្វើអ្វីទេ

តើវាអាចត្រូវប្រើប្រាស់ជាមួយវិធីសាស្រ្តការ ការពារផ្សេងៗ បានយ៉ាងដូចម្ដេច ?

អាចប្រើប្រាស់ ផើកនេះជាប់ជាមួយនឹងវិធីសាស្រ្តការការពារ ភ្នាក់ងារចំលងជំងឺទេៗទៀតបានដោយគ្មានបញ្ហា

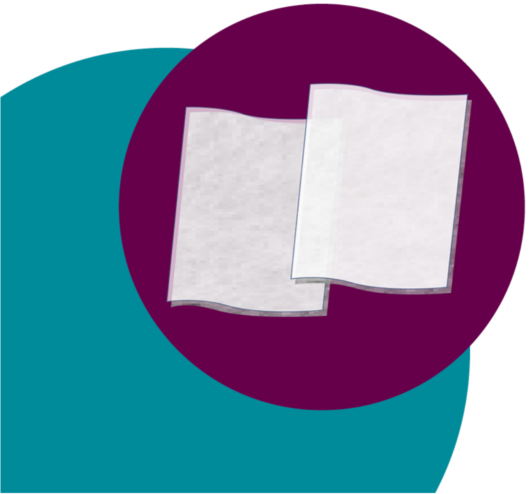

តើអ្វីខ្លះដែលត្រូវធ្វើ ?

- មុខពេលប្រើ រក្សាទុកកន្លែងត្រជាក់ និងស្ងួតផុតពីពន្លឺព្រះអាទិត្យ និងថង់ប្លាស្ទិចត្រូវបានខ្ចប់ទុករហូតដល់ថ្ងៃប្រើប្រាស់វា
- ពាក់ស្រោមដៃមុនពេលបើកកញ្ចប់ និងបើកកញ្ចប់លុះត្រាតែអ្នករៀបចំព្យួរ និងប្រើវា
- ព្យួរសន្លឹកក្រដាសនៅលើក្បាលរបស់អ្នកនៅខាងក្នុង ឬខាងក្រៅផ្ទះ ឬតាមលក្ខខណ្ឌក្នុងចំការ ឬក្នុងព្រៃ ឬក៏នៅលើដើមឈើ
- ព្យួរសន្លឹកក្រដាសនៅកន្លែងដែលមានមនុស្សចំណាយពេលច្រើននៅទីនោះ
- សន្លឹកក្រដាសត្រូវទុកដាក់ឱ្យផុតពីដៃក្មេង
- ប្រើសន្លឹកក្រដាសច្រើនតាមដែលអាចធ្វើបានទោះបីកន្លែងកំពុងប្រើមុងនិងងអង្រឹងជ្រលក់ថ្នាំ
- សំអាតដៃរបស់អ្នកជាមួយសាប៊ូ និងទឹក បន្ទាប់ពីព្យួរវា
- ផ្លាស់ប្ដូរសន្លឹកក្រដាសមួយទៀត បន្ទាប់ពីបានប្រើប្រាស់វា អស់រយពេល៣០ថ្ងៃហើយ
- យកសន្លឹកក្រដាសទៅជាមួយអ្នក ដូចជាអ្នកផ្លាស់ទីពីផ្ទះចូលចំការ ព្រៃ ឬប៉ុស្ដិ៍អភិរក្ស និងពេលត្រឡប់មកវិញ ដែលប្រសិទ្ធភាពសន្លឹកក្រដាសអាចប្រើដោយសុវត្ថិភាពក្នុងកាលទេសៈផ្សេងៗគ្នា

អ្វីខ្លះដែលមិនត្រូវធ្វើ ?

- ព្យួរបន្ទះក្រដាសផើកក្នុងកន្លែងដែលមិនមានមនុស្សនៅ (ឧទាហរណ៍ កន្លែងក្រោលសត្វនៅជាដើម)
- កុំព្យួរបន្ទះក្រដាសផើកនៅកន្លែងប៉ះផ្ទាល់នឹងពន្លឺព្រះអាទិត្យ ឬក៏ភ្លៀងក្នុងរយពេលយូរ
- ប្រើប្រាស់បន្ទះក្រដាសផើក ប្រសិនបើការវេចខ្ចប់ត្រូវបានបើកចំហមុនពេលដែលអ្នកបានរៀបចំរួចជាស្រេចក្នុងការប្រើប្រាស់វា
- កុំឱ្យបន្ទះក្រដាសផើកសើម ប្រសិនបើអ្នកកំពុងយកទៅព្រៃ ទុកដាក់ក្នុងថង់ប្លាស្ទិចការពារពីភ្លៀងសើម និងយកវាចេញពីថង់ពេលដែលអ្នករៀបចំប្រើប្រាស់វា
- កុំឱ្យវាទៅនរណាម្នាក់ក្រៅពីក្រុមគ្រួសារ ឬក្រុមអ្នកភិរក្សព្រៃឈើ

អំពីសុវត្ថិភាពនៃការប្រើប្រាស់បន្ទះ ផើក PIRK)

- **PIRK** វាគ្មានក្លិន គ្មានឆ្អួល!
- ការប៉ះផ្ទាស់ជាស្បែកអាចបង្ករជាកន្ទួល ឬក៏រមាស់បន្តិចបន្តួច
  - ប្រើប្រាស់ស្រោមដៃ ឬថង់ប្លាស្ទិច នៅពេលការកាន់វា (PIRK)
  - ប្រសិនបើអ្នកត្រូវបានរំខានពីកន្ទួល ឬរមាស់ សូមទៅពិគ្រោះនៅមណ្ឌលសុខភាពនៅក្បែរ សញ្ញាទាំងនេះនឹងបាត់ទៅវិញឆាប់ៗ
- សូមរក្សាទុកបន្ទះនេះ (PIRK) ឱ្យផុតពីដៃក្មេង
- បន្ទាប់ពីព្យួរក្រដាសផើក (PIRK) សូមលាងដៃអ្នកជាមួយនឹងសាប៊ូ និងទឹក

លក្ខខណ្ឌពិសេសនៃការប្រើប្រាស់ ថ្នាំបាញ់លើស្បែកការពារមូសខាំ (Topical Repellent)

តើអ្វីទៅថ្នាំបាញ់លើស្បែកការពារមូសខាំ ?

ស្រ្តាយរាងថ្លាដែលចេញពីកំប៉ុងប្លាស្ទីចតូចជាមួយបំពងចុចចង្អុលដៃ ដែលផ្ទុករួមមាន ២០%នៃ Picaridin ឬ ១៥% នៃ DEET

តើធ្វើថ្នាំបាញ់លើស្បែកការពារមូសខាំឡើងដើម្បីអ្វី ?

បន្ទាប់ពីការបាញ់ការពារលើស្បែកខ្លួនប្រាណ មូសត្រូវបានបណ្តេញពីការទំ និងខាំលើខ្លួនរបស់អ្នក

តើវិធីប្រើប្រាស់វាយ៉ាងដូចម្តេច ?

វាត្រូវបានបាញ់លើស្បែក ហើយវាមានប្រសិទ្ធភាព លើសពី ៨ ម៉ោង

តើវាអាចត្រូវប្រើប្រាស់ជាមួយវិធីសាស្រ្តការការពារផ្សេងបានយ៉ាងដូចម្តេច ?

អាចប្រើប្រាស់ថ្នាំនេះជាប់ជាមួយនឹងវិធីសាស្រ្តការការពារដ៏ទៃទៀតដោយគ្មានបញ្ហា

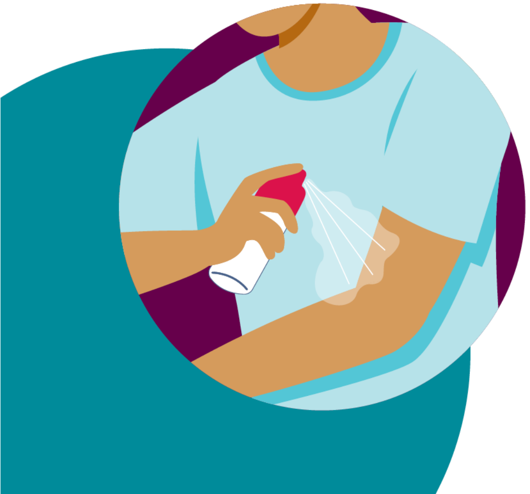

តើអ្វីខ្លះដែលត្រូវធ្វើ

- សូមរក្សាទុកកន្លែងត្រជាក់ ហើយស្ងួត មិនឱ្យត្រូវពន្លឺព្រះអាទិត្យរហូតដល់ពេលប្រើប្រាស់
- បាញ់តែទៅលើស្បែកប៉ុណ្ណោះ
- ការប្រើប្រាស់ថ្នាំនេះដែលរួមបញ្ចូលគ្នាជាមួយវិធីសាស្រ្តបង្ការមូសខាំ ដូចមានជ្រលក់ថ្នាំសម្លៀកបំពាក់ និងក្រដាសសន្លឹកព្យួរលើជញ្ជាំង
- បាញ់ថ្នាំនេះបានរហូតដល់២ដង ក្នុងមួយថ្ងៃ ពេលដែលអ្នកមិនកំពុងសំរាន ឬសម្រាកនៅលើគ្រែ ឬក្នុងមុង
- សូមរក្សាជបថ្នាំនេះផុតពីដៃក្មេងៗ

អ្វីខ្លះដែលមិនត្រូវធ្វើ

- កុំបាញ់ចូលភ្នែក ឬមាត់
- កុំផ្តល់ថ្នាំនេះទៅមនុស្សក្រៅពីគ្រួសាររបស់អ្នក ឬក៏ក្រុមអ្នកអភិរក្ស

អំពីសុវត្ថិភាព សម្រាប់ការប្រើប្រាស់ថ្នាំបាញ់ការពារមូសខាំ

- ថ្នាំបាញ់ការពារមូសខាំមានក្លិនផ្អែមបន្តិច តែក្លិននេះគឺមិនបង្ករគ្រោះថ្នាក់អ្វីឡើង
- ចៀសវាងកុំបាញ់ចំភ្នែក និងមាត់
- សូមទុកដាក់ថ្នាំនេះឱ្យផុតពីដៃក្មេង
- អាចបាញ់បាន២ដងក្នុងមួយថ្ងៃដែលមានប្រសិទ្ធភាពបាន៨ម៉ោង

លក្ខខណ្ឌពិសេសនៃប្រើប្រាស់ផលិផល សម្រាប់ការជ្រលក់ថ្នាំលើសម្លៀកបំពាក់ (Etofenprox)

អ្វីទៅជាថ្មីជ្រលក់សំលៀកបំពាក់ ?

ថ្នាំជ្រល់សម្លៀកបំបាក់អាចប្រើប្រាស់ លើសពីសម្លៀកបំពាក់ដូចជាក្រម៉ាជាដើម ក៏អាចជ្រលក់បានដែរ

តើប្រើប្រាស់ថ្នាំនេះដើម្បីអ្វី ?

ថ្នាំជ្រលក់សម្លៀកបំពាក់នេះ មិនមានន័យថាប្រើប្រាស់ដើម្បីបណ្តេញមូសទេ តែផ្ទុយទៅវិញ វាអាចសំលាប់មូសនៅពេលមូសនោះមកប៉ះពាល់នឹងសម្លៀកបំបាក់ដែលបានជ្រលក់ថ្នាំរួចរាល់ហើយ។

តើយើងប្រើប្រាស់វារបៀបណា ?

សម្លៀកបំពាក់ដែលបានបាញ់ ឬជ្រលក់រួចរាល់ហើយ គឺអាចប្រើប្រាស់ ស្លៀកពាក់បានដូច សម្លៀកបំពាក់ដែលមិនបានប្រើប្រាស់ថ្នាំនោះដែរ។ សម្លៀកបំពាក់ដែលបានជ្រលក់ាឬបាញ់ជាមួយ etofenproxរួចគឺអាចបោកគក់ដល់ទៅ២៥ដង

តើវាអាចប្រើប្រាស់រួមគ្នាជាមួយសំភារៈការពារមូសដទៃទៀតបានដែរឬទេ ?

គឺយើងអាចប្រើប្រាស់ជាមួយសំភារៈការពារមូសដទៃទៀតបានដោយងាយ និងមិនមានបញ្ហាដែរ។

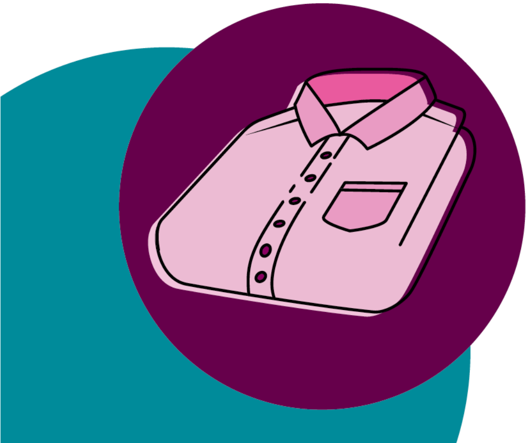

តើអ្វីខ្លះដែលត្រូវធ្វើ

- ស្លៀកពាក់សម្លៀកបំពាក់ដែលជ្រលក់ថ្នាំហើយ គឺពាក់ធម្មតាដូចខោអាវដទៃទៀតផងដែរ
- អ្នកត្រូវប្រាកដថាត្រូវតែស្លៀកពាក់សម្លៀកបំបាក់ដែលជ្រលក់ថ្នាំរួចគឺពាក់រាល់ការចូលក្នុងព្រៃ ពាក់នៅខាងក្រៅកន្លែងផ្សេង មិនបាច់ស្លៀកពាក់នៅនៅពេលគេងនៅក្នុងមុខជ្រលក់ថ្នាំ និង មុខអង្រឹងនោះទេ
- សម្លៀកបំពាក់ដែលបានជ្រលក់ថ្នាំហើយ មិនតំរូវអោយយើងមានលក្ខ័ណការពារទុកដាក់ ដូចទៅនឹងសម្លៀកបំបាក់ផ្សេងទៀតនោះទេ
- ត្រូវធានាថាទឹកដែលបោកគក់ខោអាវដែលយើងបានជ្រលក់ថ្នាំនៅត្រូវចាក់ចោលនៅឆ្ងាយពីប្រភពទឹកប្រើប្រាស់ របស់មនុស្សនិង សត្វ
- បើសម្លៀកបំពាក់ដែលជ្រលក់រួចថ្មីមានខ្លិនមិនល្អ អ្នកអាចយកទៅបោកគក់ម្តងមុនស្លៀកពាក់បាន។

អ្វីខ្លះដែលមិនត្រូវធ្វើ

- មិនអនុញ្ញាតអោយមនុស្សនៅខាងក្រៅគ្រួសាររបស់អ្នក ឬ អ្នកភិរក្សប្រើប្រាស់សម្លៀកបំពាក់ដែលជ្រលក់ថ្នាំរួចរបស់អ្នកនោះទេ។ ពួកគាត់មិនដែលទទួលបានការណែនាំវិធីការប្រើប្រាស់ដូចអ្នកនោះទេ
- កុំបោកសម្លៀកបំពាក់តាមប្រឡាយ ឬស្ទឹង ឬក៏ប្រភពទឹកផ្សេងៗដែលវាអាចជាទឹកសម្រាប់បរិភោគ ឬជម្រកសត្វក្នុងទឹក។

អំពីសុវត្ថិភាពសម្រាប់ថ្នាំជ្រលក់សម្លៀកបំពាក់

- ការជ្រលក់ថ្នាំក្នុងសម្លៀកបំពាក់វាអាចមានក្លិន ហើយក្លិននេះវាមិនបង្កភាពគ្រោះថ្នាក់ទេ និងវានឹងរលាយនៅពេលស្លៀកពាក់វា និងបោកសម្លៀកបំពាក់
- អ្នកនឹងទទួលការកន្ទួល ឬរមាស់បន្តិចបន្តួចពេលអ្នកស្លៀកពាក់ជ្រលក់ថ្នាំជាលើកដំបូង ប៉ុន្តែអ្នកអាចបោកសម្លៀកបំពាក់នេះវាអាចជួយឱ្យមានការបន្ថយនូវភាពរំខាននេះ
- ការបោកសម្លៀកបំពាក់ដែលបានជ្រលក់ថ្នាំរួចគឺត្រូវធ្វើឡើងតាមការណែនាំ
