# Supplementary material for: Evaluating the acceptability and feasibility of new mosquito bite prevention tools in a “forest pack” to support malaria elimination in Cambodia
Source: Malar J. 2025 Nov 27;24:443. doi: 10.1186/s12936-025-05682-2 (PMC12715958; doi:10.1186/s12936-025-05682-2)
Supplement: Supplementary file 3 — Additional file3 (PDF 74 KB) [file 12936_2025_5682_MOESM3_ESM.pdf]

Introduction and Hello

Hello and how have you been since we last met?

Life throws plenty of challenges our way and it is my hope that by keeping you better protected from mosquito bites, you will have less to worry about.

How can we thrive as a community if we are unwell?

How can our families prosper if we fall ill?

How can rangers protect the forests if they get sick?

The easiest way to free your mind from these worries is to do everything you can to prevent mosquito bites. This means using all three of products we gave you last month for full protection.

Have you had any challenges using the 3 new products I gave you since we last met?

Treated Clothing

Let’s talk about the treated clothing: have you faced any challenges wearing the clothing we treated for you every day?

Does anyone else in the group (or household) have an idea about how to deal with this challenge?

Remember that wearing the clothing we treated for you is a great way to protect yourself from mosquito bites in any situation, including when you are outside. Think of it as your **super-suit**. Gain power against mosquitos by wearing treated clothing!

Treated Sheets

Let’s talk about the repellent sheets: have you faced any challenges hanging these every day?

Remember that hanging the repellent sheets can strengthen your protection from mosquitoes even in situations where bed and hammock nets can’t be used.

And don’t forget that whereas using 1 of the 3 products we gave you is smart, using all 3 is the smartest as it gives you more power to fight off mosquitoes.

Does anyone in the group (or household) have an idea about how to deal with this challenge?

Topical Repellent

Let’s talk about the body repellent: have you faced any challenges using repellent every day?

Remember that body repellent can protect the parts of your body that may not be fully protected by the other tools we gave you.

What if your shirt sleeves get wet when you are in the forest, and you roll-them up for comfort while you are working? If you have applied body repellent already that morning before going to the forest, you’re better protected!

Does anyone in the group (or household) have an idea about how to deal with this challenge?

Using All Products

Have you wondered if you need to use all 3 of the products I gave you?

That's a little like asking how much petrol you need to put in your motorbike. By using all 3 of the products I gave you every day, you're filling your mosquito-fighting tank. Just like you would never go to the forest with less petrol than you need in your tank, why would you use less than full protection from mosquito bites?

Does anyone in the group (or household) have an idea about how to remember to use all 3 products every day?

One thing you can do is ask someone in your house, forest or ranger group to remind you! Groups that help each other use maximum protection are going to have more strength!

<u>Questions (1)</u>

We've heard others in the community discussing some interesting questions about the products they've started using for more complete protection from mosquito bites. What are your thoughts about these common questions?

Are the repellent sheets safe?

Yes! The repellent sheets are very safe to have around you. They don't have any smell and don't make you feel nauseous. Just remember to use gloves when you touch the sheet.

Mosquito bites are annoying--but they won't cause me any serious harm, so why should I use more than one prevention tool?

Wrong! Mosquitoes can carry dangerous diseases like malaria and dengue. The more tools you use to protect yourself from mosquito bites, the safer you will be from these diseases.

Questions (2)

What if it rains on the treated sheets, can I still use them?

It's best to keep your treated sheets dry and away from rain. If they do get wet, you can still use them, but they may not work quite as well as if they were dry.

Can I share my treated clothing with my sister in the next village if she's going to her farm and needs extra protection?

Your treated clothing is only for you to use. You need it to protect yourself from mosquitoes, and only you have had the proper training to understand how to use them properly and how to be safe when using them.

What questions do you hear about the products? Do you have any questions you'd like us to try to answer today or in our next visit?

Wrap Up and P.L.A.N.

I’m so glad we had a chance to talk about your experience using these new products. You’re so much better protected today than you were before! Going forward, **don’t forget to P.L.A.N**:

Prepare to carry and use all prevention products every day, even when you go to the forest
Leave all worries behind, knowing that you have full protection from mosquito bites
Always encourage others in your family or ranger group to use all 3 products, but
Never share the products outside your family or ranger group

Thanks for talking with me today, I learned a lot and I’m looking forward to visiting you again soon so that I can hear about the difference these 3 products are making in your house and community.
