# Supplementary material for: Evaluating the acceptability and feasibility of new mosquito bite prevention tools in a “forest pack” to support malaria elimination in Cambodia
Source: Malar J. 2025 Nov 27;24:443. doi: 10.1186/s12936-025-05682-2 (PMC12715958; doi:10.1186/s12936-025-05682-2)
Supplement: Supplementary file 4 — Additional file4 (PDF 72 KB) [file 12936_2025_5682_MOESM4_ESM.pdf]

<u>សេចក្តីផ្តើម និងជំរាបសួរ</u>

- ជំរាបសួរ ហើយតើអ្នកសុខសប្បាយជាទេតាំងពីយើងជួបគ្នាលើកមុន ?

- ត្រូវតែធ្វើអោយជីវិតយើងជៀសចេញពីបញ្ហាទាំងឡាយ ហើយវាជាក្តីសង្ឃឹមរបស់ខ្ញុំធ្វើយ៉ាងណាដើម្បីការការពារអ្នកឱ្យកាន់តែប្រសើរឡើងពីការមូសខាំ ហើយអ្នកនឹងមិនសូវមានការព្រួយបារម្ភនោះទេ។

- តើយើងអាចក្លាយជាភូមិឬសហគមន៍រីកចម្រើនបានយ៉ាងដូចម្តេច បើយើងម្នាក់ៗមានជម្ងឺជាប់ខ្លួន ?

- តើគ្រួសារយើងអាចរីកចម្រើនបានដោយរបៀបណា បើយើងធ្លាក់ខ្លួនឈឺ ឬមានជម្ងឺជាប់ខ្លួន ?

- តើមន្ត្រីអភិរក្សព្រៃអាចការពារព្រៃឈើដោយរបៀបណា បើពួកគាត់ឈឺ ឬមានជម្ងឺជាប់ខ្លួន ?

- មធ្យោបាយដ៏ងាយស្រួលបំផុតដើម្បីជៀសពីការព្រួយបារម្ភទាំងនេះគឺធ្វើអ្វីគ្រប់យ៉ាងដែលអ្នកអាចធ្វើបានដើម្បីការពារកុំឱ្យមូសខាំ។ នេះមានន័យថាការប្រើប្រាស់ផលិតផលទាំង៣ដែលយើងបានផ្តល់ឱ្យអ្នកកាលពីខែមុនសម្រាប់ការការពារមូសខាំពេញលេញ។

- តើអ្នកមានបញ្ហាអ្វីខ្លះក្នុងការប្រើផលិតផលថ្មីទាំង៣ដែលយើងបានផ្តល់ឱ្យអ្នកតាំងពីយើងបានជួបលើកមុនដែរឬទេ ?

<u>សំលៀកបំពាក់ជ្រលក់ឬបាញ់ថ្នាំការពារមូសខាំ</u>

- និយាយអំពីសម្លៀកបំពាក់ដែលបានជ្រលក់ឬបាញ់ថ្នាំ៖ តើអ្នកមានបញ្ហាប្រឈមក្នុងការស្លៀកពាក់សម្លៀកបំពាក់ទាំងនោះជារៀងរាល់ថ្ងៃដែរឬទេ ?

- តើមាននរណាម្នាក់ផ្សេងទៀតនៅក្នុងក្រុម ឬស្នាក់ការ ឬគ្រួសារ មានវិធីក្នុងការដោះស្រាយបញ្ហាប្រឈមនេះដែរឬទេ ?

- សូមចងចាំថាការស្លៀកពាក់សម្លៀកបំពាក់ដែលបានជ្រលក់ឬបាញ់ថ្នាំគឺជាវិធីដ៏ល្អមួយដើម្បីការពារខ្លួនអ្នកពីមូសខាំក្នុងគ្រប់ស្ថានភាព រួមទាំងពេលអ្នកចេញក្រៅផ្ទះ ស្នាក់ការ ឬភូមិផងដែរ។ ចូរគិតថាវាជា**<u>ឈុតដ៏អស្ចារ្យ</u>**របស់អ្នក។ ទទួលបានថាមពលដ៏ខ្លាំងខ្លាប្រឆាំងនឹងមូស ដោយការស្លៀកសំលៀកបំពាក់ជ្រលក់ឬបាញ់ថ្នាំ។

<u>**បន្ទះថ្នាំការពារតាមលំហអកម្ម (ភើក)**</u>

- និយាយអំពីបន្ទះឬសន្លឹកថ្នាំការពារតាមលំហអកម្ម (ភើក)៖ តើអ្នកមានបញ្ហាប្រឈមនឹងការព្យួរវាជារៀងរាល់ថ្ងៃដែរឬទេ?

- សូមចងចាំថាការព្យួរបន្ទះឬសន្លឹកថ្នាំភើកអាចពង្រឹងការការពាររបស់អ្នកពីមូសខាំ សូម្បីតែនៅក្នុងស្ថានភាពដែលគ្មានគ្រែ គ្មានមុងប្រើក៏ដោយ។

- ហើយកុំភ្លេចថា ការប្រើប្រាស់ផលិតផល១ក្នុងចំណោមផលិតផលទាំង៣ដែលយើងផ្តល់ឱ្យអ្នកគឺជាការឆ្លាតវៃ ហើយបើការប្រើទាំង៣វិញគឺជាការឆ្លាតវៃបំផុតព្រោះវាផ្តល់ថាមពលកាន់តែខ្លាំងក្នុងការប្រយុទ្ធប្រឆាំងពីមូសខាំ។

- តើមាននរណាម្នាក់នៅក្នុងក្រុម ឬស្នាក់ការ ឬគ្រួសារអ្នក មានវិធីក្នុងការដោះស្រាយបញ្ហាប្រឈមនេះដែរឬទេ?

<u>ថ្នាំបាញ់ ឬលាបលើស្បែកការពារមូសខាំ</u>

- និយាយអំពីថ្នាំបាញ់ ឬលាបលើស្បែកការពារមូសខាំ៖ តើអ្នកមានបញ្ហាប្រឈមនឹងការប្រើប្រាស់វាជារៀងរាល់ថ្ងៃដែរឬទេ ?

- សូមចងចាំថាថ្នាំបាញ់ ឬលាបលើស្បែកអាចការពារផ្នែកនៃរាងកាយរបស់អ្នកបាន បើផលិតផលផ្សេងទៀតដែលយើងផ្តល់ឱ្យអ្នកមិនបានការពារពីមូសខាំពេញលេញនោះទេ។

- ចុះបើដៃអាវរបស់អ្នកសើមពេលអ្នកនៅក្នុងព្រៃ ហើយអ្នកលាត់ដៃអាវឡើងដើម្បីអោយស្រួលពេលអ្នកកំពុងធ្វើការ ? បើអ្នកបានបាញ់ឬលាបថ្នាំការពារលើខ្លួនរួចហើយនៅពេលព្រឹកនោះមុនចូលព្រៃ មានន័យថាអ្នកបានការពារខ្លួនល្អហើយ !

- តើមាននរណាម្នាក់នៅក្នុងក្រុម ឬស្នាក់ការ ឬគ្រួសារអ្នក មានវិធីក្នុងការដោះស្រាយបញ្ហាប្រឈមនេះដែរឬទេ ?

<u>ការប្រើប្រាស់ផលិតផលការពារមូសខាំទាំង៣</u>

- តើអ្នកមានឆ្ងល់ថា អ្នកត្រូវតែប្រើផលិតផលទាំង៣ដែលខ្ញុំបានផ្តល់ឱ្យអ្នកដែរទេ ?

- វាដូចជាការសួរថាតើអ្នកត្រូវការសាំងប៉ុន្មានដើម្បីចាក់ម៉ូតូរបស់អ្នក។ ដោយប្រើផលិតផលទាំង៣ដែលយើងបានផ្តល់ឱ្យអ្នកជារៀងរាល់ថ្ងៃ វាមានន័យថាអ្នកកំពុងបំពេញធុងសម្រាប់ការពារមូសខាំ។ វាដូចជាអ្នកទៅព្រៃមិនដែលមានសាំងតិចជាងតម្រូវការក្នុងធុងសាំងម៉ូតូរបស់អ្នកទេ ហេតុអ្វីបានជាអ្នកប្រើតិចជាងការការពារពេញលេញពីមូសខាំ ?

- តើមាននរណាម្នាក់នៅក្នុងក្រុម ឬស្នាក់ការ ឬគ្រួសារអ្នក មានវិធីចងចាំក្នុងការប្រើផលិតផលទាំង៣នេះជារៀងរាល់ថ្ងៃដែរឬទេ ?

- រឿងមួយដែលអ្នកអាចធ្វើបានគឺសុំនរណាម្នាក់ដែលជាសមាជិកគ្រួសារ ឬក្រុមចូលព្រៃជាមួយគ្នា ឬក្រុមអភិរក្សព្រៃស្នាក់ការជាមួយគ្នាដើម្បីរំលឹកអ្នក! ក្រុមដែលជួយរំលឹកគ្នាប្រើផលិតផលការពារមូសខាំអតិបរិមានឹងមានថាមពលនិងកម្លាំងខ្លាំងខ្លា!

<u>**សំនួរទី១**</u>

- យើងបានឮអ្នកផ្សេងទៀតនៅក្នុងសហគមន៍ពិភាក្សាអំពីសំណួរគួរឱ្យចាប់អារម្មណ៍មួយចំនួនអំពីផលិតផលដែលពួកគេបានចាប់ផ្តើមប្រើប្រាស់សម្រាប់ការការពារពេញលេញដើម្បីការពារពីមូសខាំ។ តើអ្នកយល់យ៉ាងណាចំពោះសំណួរទូទៅទាំងនេះ?

- **តើបន្ទះឬសន្លឹកថ្នាំការពារតាមលំហអកម្ម (ភើក) មានសុវត្ថិភាពដែរឬទេ?**
បាទ/ចាសពិតជាមានសុវត្ថិភាព! បន្ទះឬសន្លឹកថ្នាំការពារតាមលំហអកម្ម (ភើក) គឺមានសុវត្ថិភាពខ្លាំងណាស់សំរាប់អ្នកប្រើប្រាស់។ វាគ្មានក្លិន ហើយមិនធ្វើឱ្យអ្នកមានអារម្មណ៍ចង់ក្អួតទេ។ សូមចងចាំថាអ្នកត្រូវប្រើស្រោមដៃនៅពេលអ្នកប៉ះបន្ទះឬសន្លឹកថ្នាំនោះ។

- **មូសខាំគឺគួរឱ្យរំខាន ប៉ុន្តែវានឹងមិនធ្វើឱ្យខ្ញុំមានគ្រោះថ្នាក់អ្វីធ្ងន់ធ្ងរនោះទេ ដូច្នេះហេតុអ្វីបានជាខ្ញុំគួរប្រើផលិតផលការពារច្រើនជាងមួយ?**
ខុស! មូសអាចចម្លងជំងឺគ្រោះថ្នាក់ដូចជាគ្រុនចាញ់ និងគ្រុនឈាម។ អ្នកនឹងមានសុវត្ថិភាពពីជំងឺទាំងនេះបើអ្នកប្រើផលិតផលការពារមូសខាំកាន់តែច្រើន។

<u>**សំនួរទី២**</u>

- **ចុះបើភ្លៀងធ្លាក់លើបន្ទះឬសន្លឹកថ្នាំការពារអកម្ម (ភើក) តើខ្ញុំនៅតែអាចប្រើវាបានដែរឬទេ?**
  វាជាការល្អបំផុតក្នុងការរក្សាបន្ទះឬសន្លឹកថ្នាំការពារអកម្ម (ភើក) របស់អ្នកឱ្យស្ងួត និងឆ្ងាយពីទឹកភ្លៀង។ ប្រសិនបើវាសើមអ្នកនៅតែអាចប្រើវាបានប៉ុន្តែពួកវាប្រហែលជាមិនមានប្រសិទ្ធភាពល្អដូចស្ងួតទេ។

- **តើខ្ញុំអាចអោយសម្លៀកបំពាក់ដែលជ្រលក់ឬបាញ់ថ្នាំរបស់ខ្ញុំអោយទៅបងប្អូនរបស់ខ្ញុំនៅភូមិជិតហ្នឹងបានទេ ប្រសិនបើគាត់ត្រូវទៅចំការឬវាលស្រែរបស់គាត់ ហើយត្រូវការការការពារបន្ថែមពីមូសខាំ?**
  សម្លៀកបំពាក់របស់អ្នកគឺសម្រាប់តែអ្នកប្រើប៉ុណ្ណោះ។ អ្នកត្រូវការវាដើម្បីការពារខ្លួនអ្នកពីមូស ហើយមានតែអ្នកប៉ុណ្ណោះដែលបានទទួលការបណ្តុះបណ្តាលត្រឹមត្រូវនិងយល់ពីរបៀបប្រើប្រាស់វាឱ្យបានត្រឹមត្រូវ និងមានសុវត្ថិភាពនៅពេលប្រើវា។

- តើអ្នកឮសំណួរអ្វីខ្លះអំពីផលិតផលទាំងនេះ? តើអ្នកមានសំណួរណាមួយដែលអ្នកចង់ឱ្យយើងឆ្លើយនៅថ្ងៃនេះ ឬនៅពេលជួបលើកក្រោយដែរឬទេ?

<u>រំលឹក និងដាក់ផែនការ (P.L.A.N)</u>

ខ្ញុំរីករាយណាស់ដែលយើងមានឱកាសនិយាយអំពីបទពិសោធន៍របស់អ្នកក្នុងការប្រើផលិតផលការពារមូសខាំថ្មីទាំងនេះ។ ថ្ងៃនេះអ្នកបានការពារមូសបានប្រសើរជាងមុន! ទៅមុខកុំភ្លេច រៀបចំផែនការ (P.L.A.N)

P: **ត្រៀមខ្លួន**៖ ត្រៀមខ្លួននឹងប្រើប្រាស់គ្រប់ផលិតផលការពារមូសខាំជារៀងរាល់ថ្ងៃសូម្បីចូលព្រៃក៏ដោយ
L: **បោះចោល**៖ បោះចោលភាពកង្វល់ទាំងឡាយដោយដឹងថាអ្នកមានការការពារពេញលេញពីមូសខាំ
A: **តែងតែ**៖ តែងតែលើកទឹកចិត្តសមាជិកក្នុងគ្រួសារ ឬក្រុមអភិរក្សព្រៃរបស់អ្នកឱ្យប្រើផលិតផលការពារមូសទាំង៣ ក៏ប៉ុន្តែ
N: **ហាម ឬកុំ**៖ កុំចែករំលែកផលិតផលការពារមូសខាំទាំងនេះទៅអ្នកផ្សេង

អរគុណសម្រាប់ការនិយាយជជែកជាមួយខ្ញុំនៅថ្ងៃនេះ ខ្ញុំបានរៀននិងយល់ច្រើន ហើយខ្ញុំទន្ទឹងរង់ចាំមកជួបអ្នកម្តងទៀតក្នុងពេលឆាប់ៗនេះដើម្បីអោយខ្ញុំដឹងអំពីភាពខុសគ្នានៃផលិតផលទាំង៣នេះនៅក្នុងផ្ទះរបស់អ្នក ក៏ដូចជានៅភូមិឬសហគមន៍របស់អ្នកដែរ។
