# Supplementary material for: Evaluating the acceptability and feasibility of new mosquito bite prevention tools in a “forest pack” to support malaria elimination in Cambodia
Source: Malar J. 2025 Nov 27;24:443. doi: 10.1186/s12936-025-05682-2 (PMC12715958; doi:10.1186/s12936-025-05682-2)
Supplement: Supplementary file 5 — Additional file5 (PDF 5485 KB) [file 12936_2025_5682_MOESM5_ESM.pdf]

HOW TO FULLY PROTECT YOURSELF FROM MOSQUITO BITES

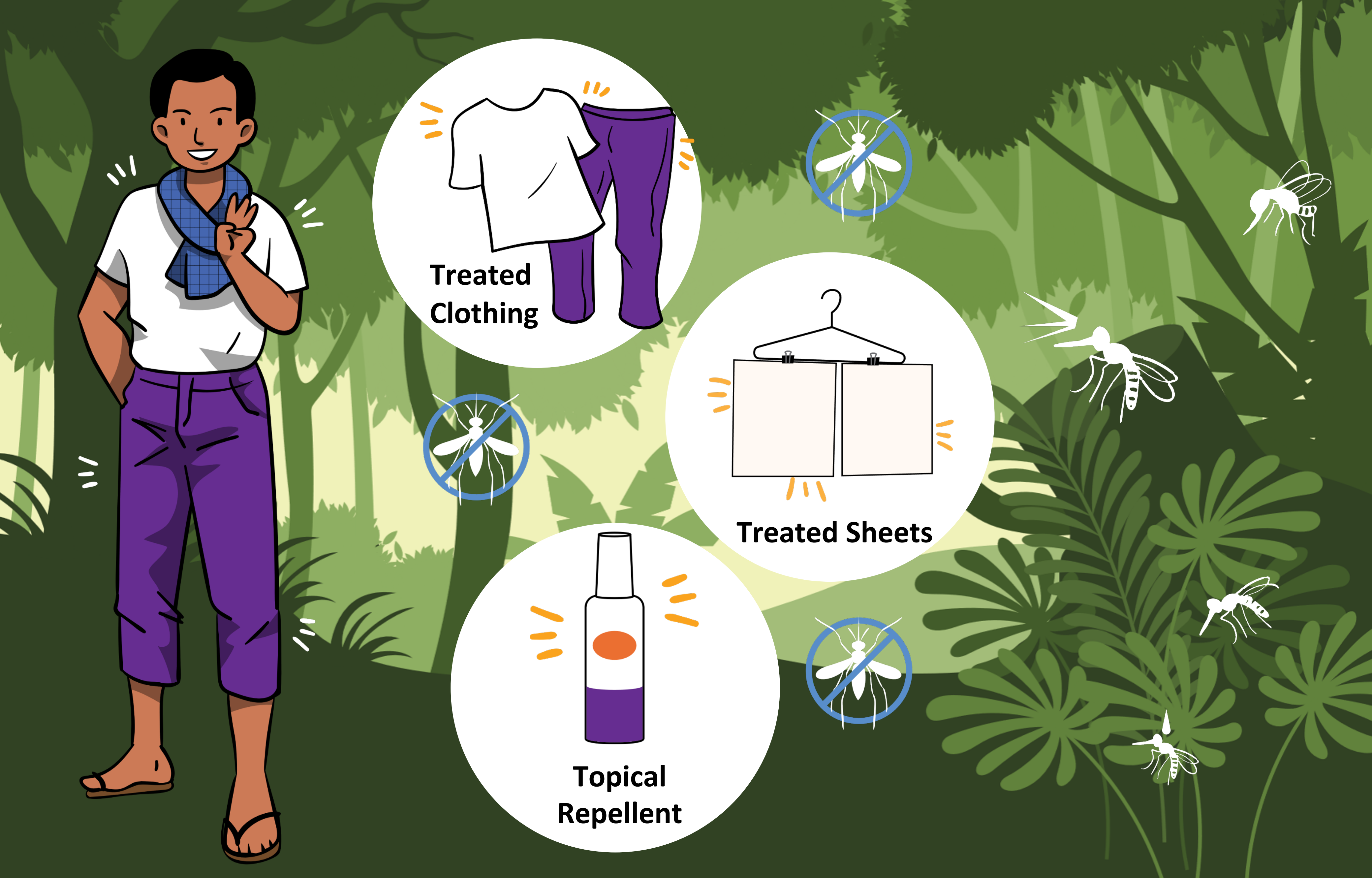

Talking points for Village Malaria Workers, Mobile Malaria Workers, Village Chiefs, and head of Indigenous peoples:

Hello and how have you been since we last met?

I’m here to provide new products, and to remind you about the **benefits of using all of the 3 new products.**

Life comes with uncertainty and risks. The good news is that by using these 3 new products, you can reduce one of those risks and protect yourself more fully from mosquito bites and related illnesses.

Less risk, means you and your family are more likely to prosper. **Let’s talk more about how to use all 3 products for the full power you need to stay free of mosquito bites!**

Both of these images show where to use **treated clothing**

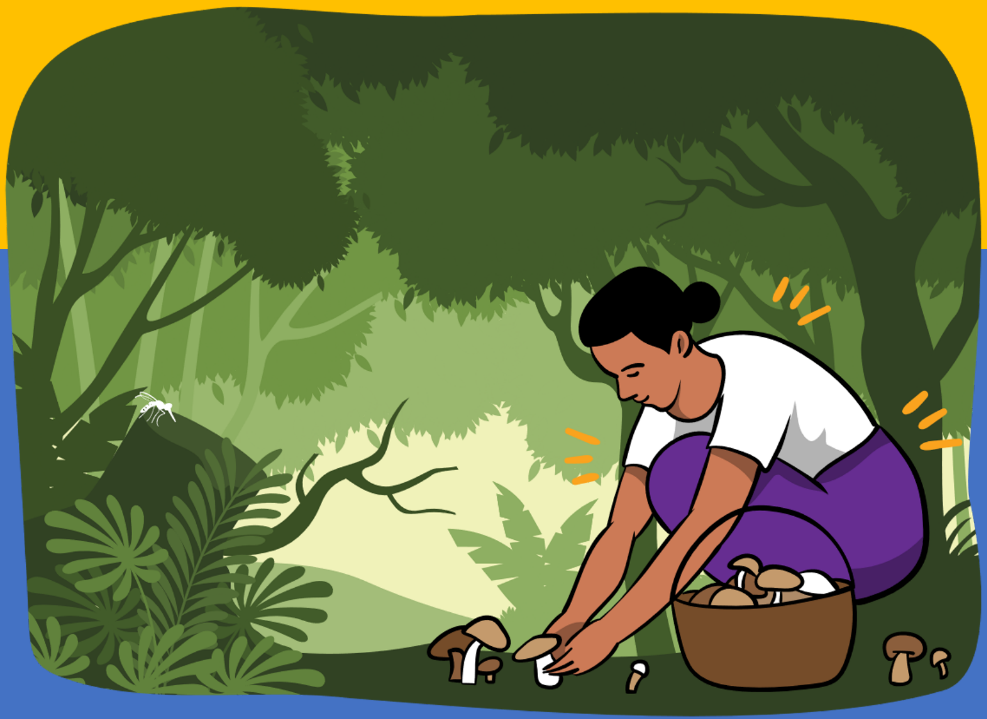

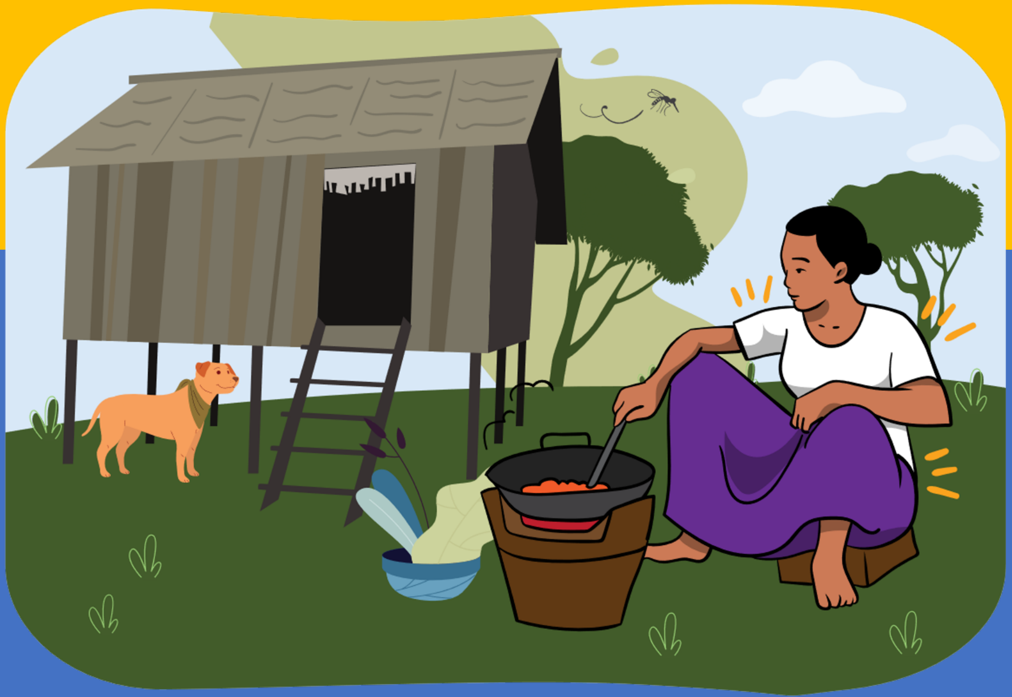

The best way to use treated clothing is to wear your treated clothing as much as possible, especially outside–when you visit the forest and when you are near your home.

BOTH of these illustrations show the recommended ways you can use your treated clothing–so that you are protected in any setting where you may be at risk of mosquito bites.

Some people mistakenly think that the treating clothing can only be used when people go to the forest, but **the best way to have the most protection is to wear this clothing anytime you are outside.**

Think of it as your **super-power suit**!

If the smell of freshly treated clothing bothers you, wash once or twice to remove the odor. Treated clothing is effective up to 25 washes!

Do you have any questions?

Which of these images shows the best way to use repellent sheets?

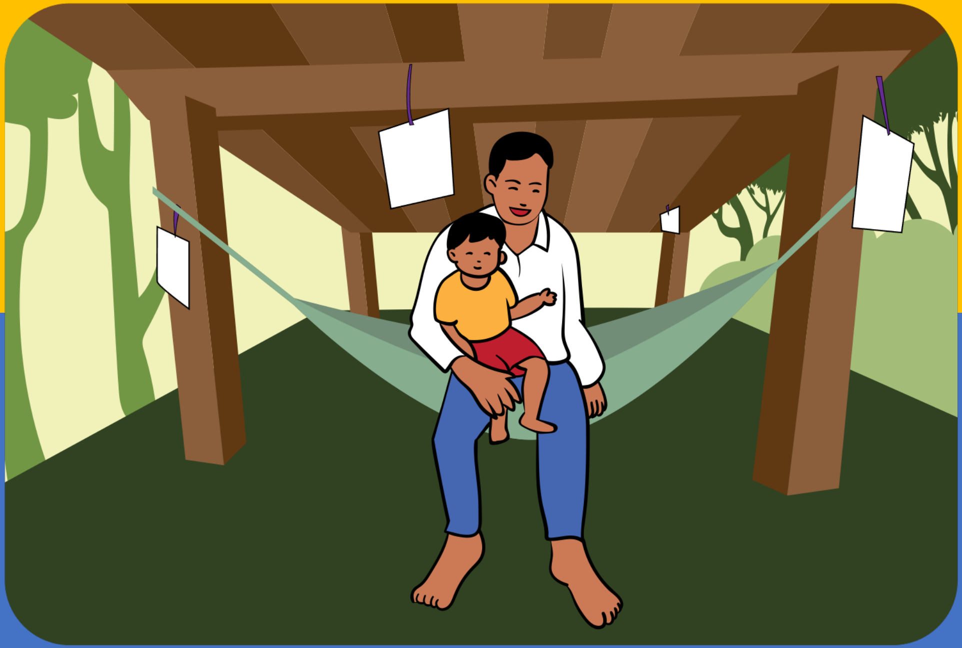

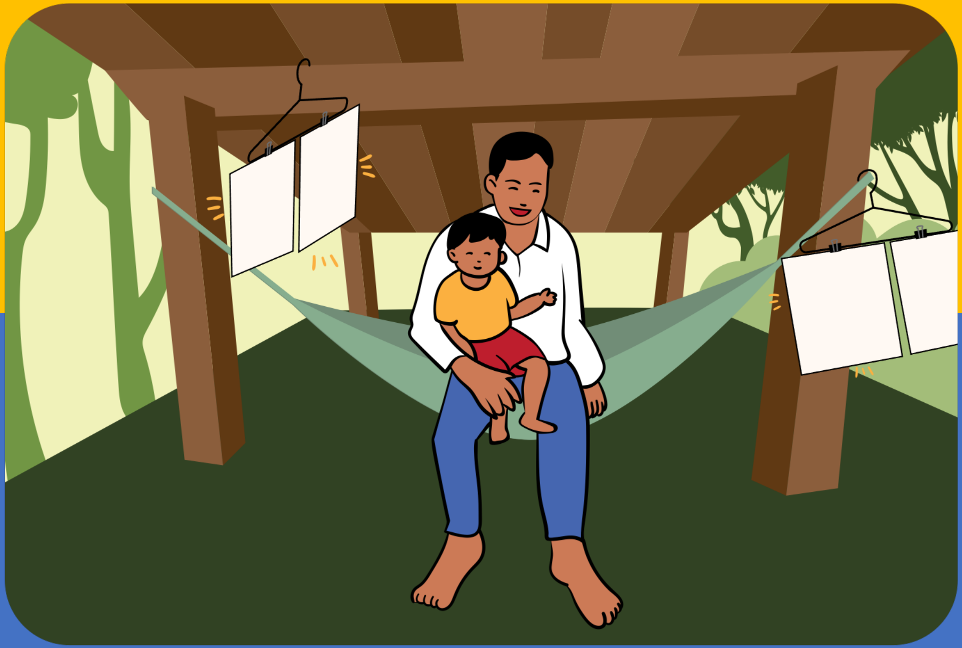

The best way to use repellent sheets is to hang the sheets in pairs (of 2!)

The image on the right hand side shows the correct way to hang the sheets – 2 sheets together
(instead of single sheets). Hang them wherever you spend time and take them to the forest with you.

Hang the sheets at least 2 meters off the ground, and don’t let children touch them. The treated sheet has no smell, but already has the repellent inside it – no need to spray it with topical repellent!
Always wear gloves when you touch the treated sheets!

Do you have any challenges using the sheets as recommended?

Which of the following images shows the best way to use repellent?

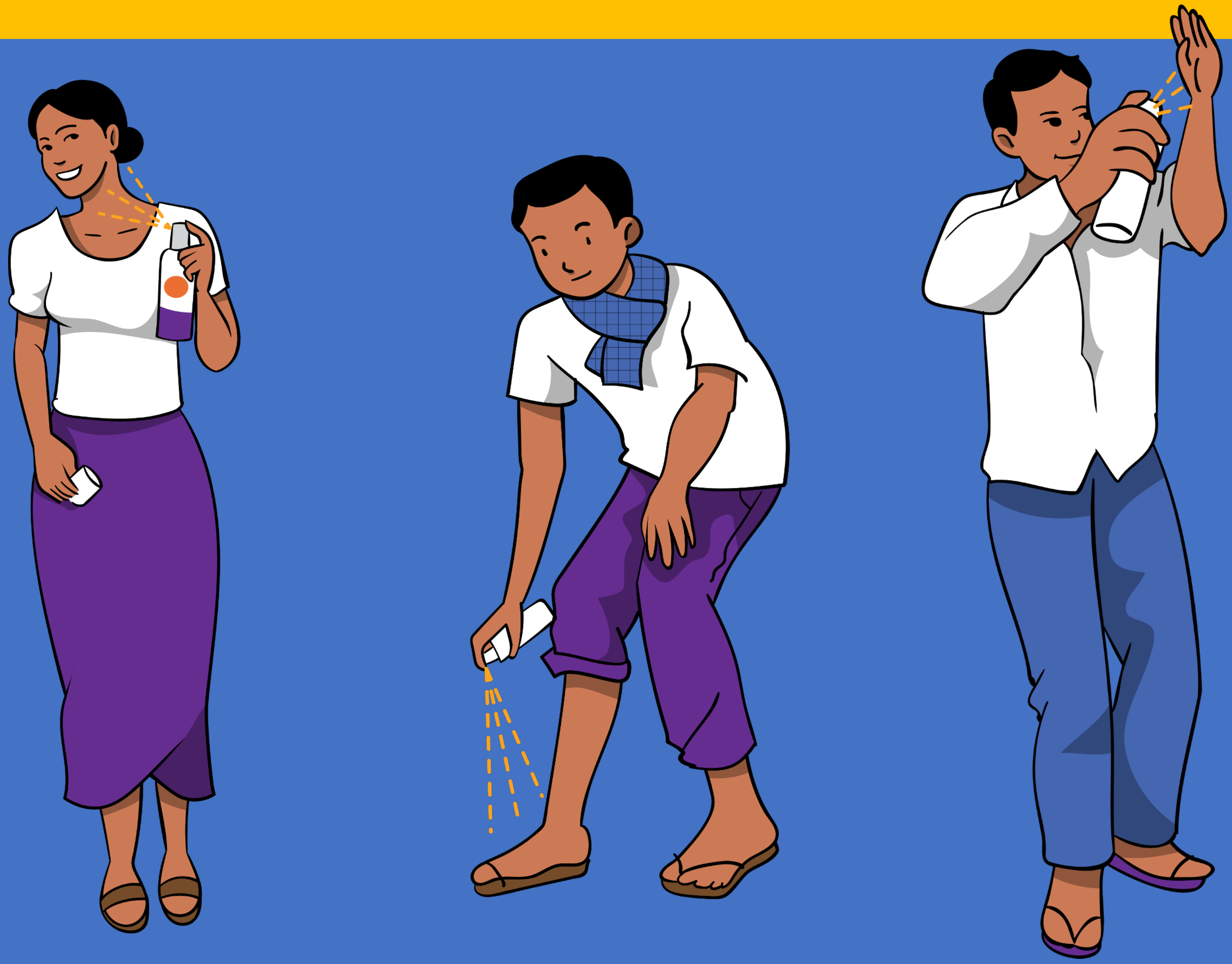

The best way to use personal repellent is to spray the parts of your body that may be exposed to mosquito bites.

All of these images show the importance–for men and women–to use body repellent every day to protect the parts of your body that may not be fully protected by the other tools you received.

Even if you are wearing longsleeves or pants, you should spray your arms and legs (under your clothing) so that you are well-protected – even at home.

Only spray the repellent on your skin – not on bed nets or hammock nets, blankets/pillows, or on the treated sheets.

Do you have any questions about body repellent?

Which of these products do you think it is important to use if you also have a treated bed or hammock net?

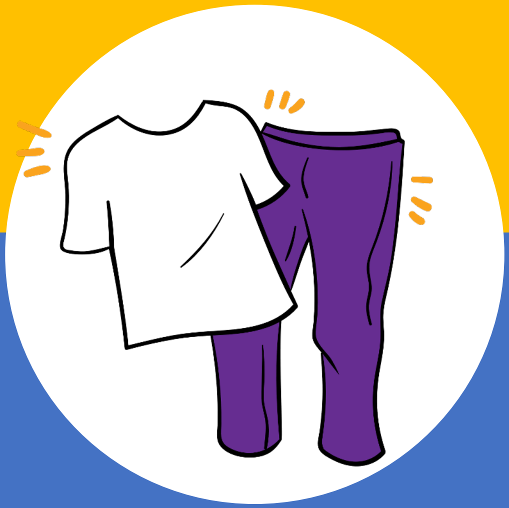

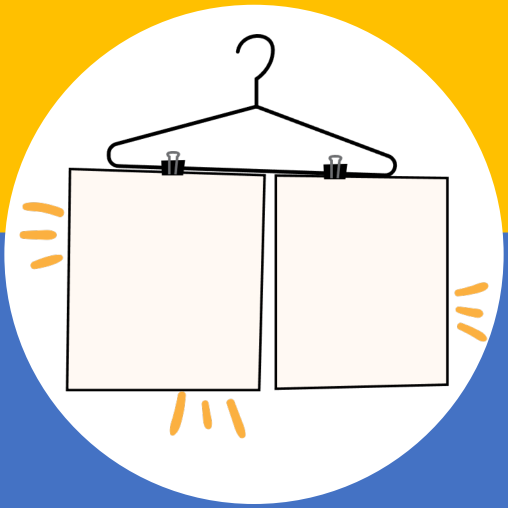

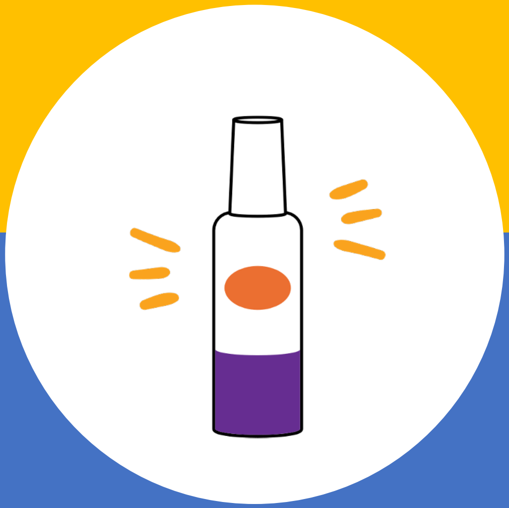

Actually, the best way for the most protection and not worry about mosquito bites and related illness is to use ALL THREE, together with a treated net! Just like you would never start a planting season with less water and fertilizer than you need for the best crops, why would you use less than full protection from mosquito bites?

Have you asked people you stay or work with to help you to remember to bring and use all 3 products with you when you are outside?

Other houses I visit tell me that when they support each other as a group, they are more likely to avoid mosquito bites by using all 3 of these products every day.

What can you do to remember to use all 3 products every day between now and when I visit you next month?

Families and ranger groups that help each other use maximum protection are going to have more strength!

Truth or Myth?

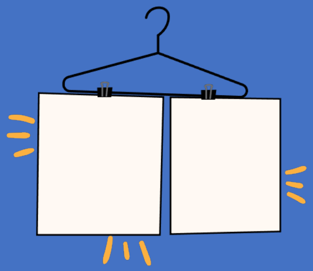

Repellent sheets are safe to use for people of all ages

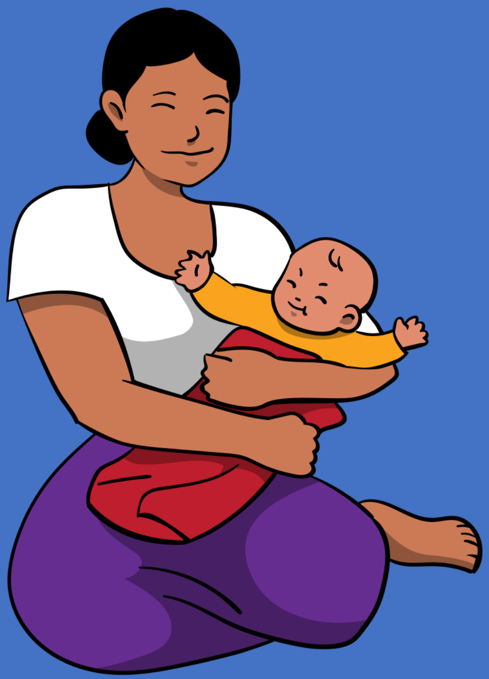

TRUTH!

The repellent sheets are very safe to have around you. They don’t have any smell and don’t make you feel nauseous. Just remember to use gloves when you touch the sheet.

Truth or Myth?

Mosquito bites are annoying, but they can't cause serious illness

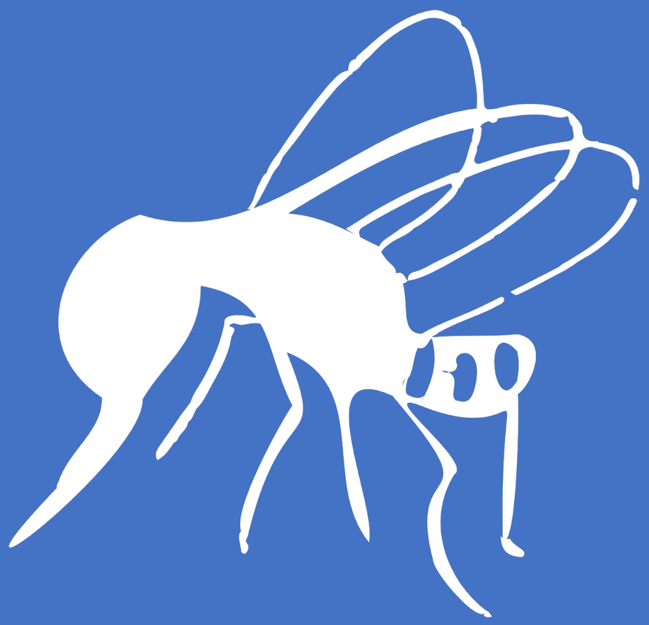

FALSE!

The truth is that mosquitoes can carry dangerous diseases like malaria and dengue. The more tools you use to protect yourself from mosquito bites, the safer you will be from these diseases.

Truth or Myth?

Each family only needs 1 piece of treated clothing?

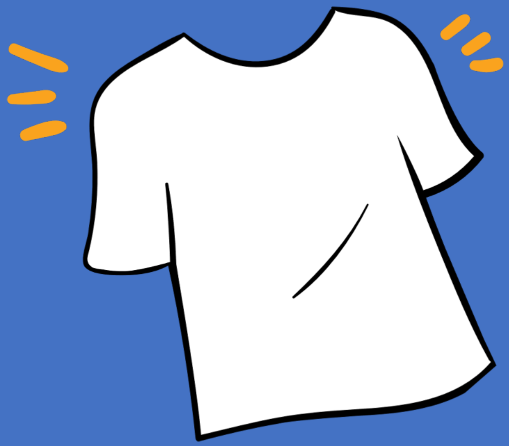

VS

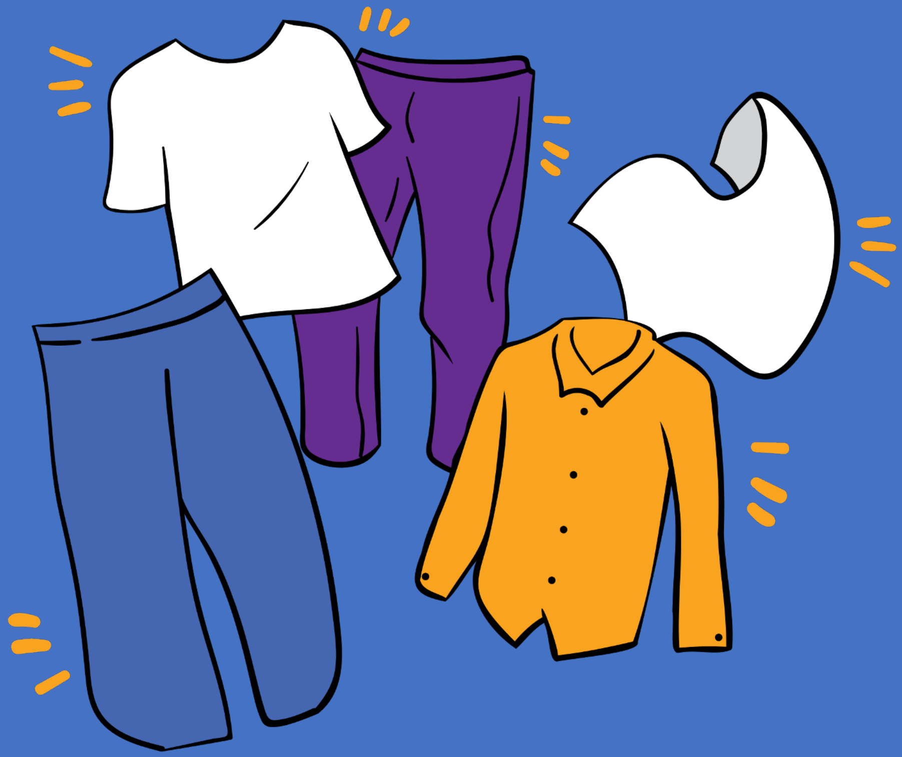

FALSE!

The truth is that each PERSON should receive 4-5 pieces of treated clothing, so that you can wear different treated clothing every day.

Truth or Myth?

It's okay to share my treated clothing with my sister in the next village if she's going to her farm and needs extra protection

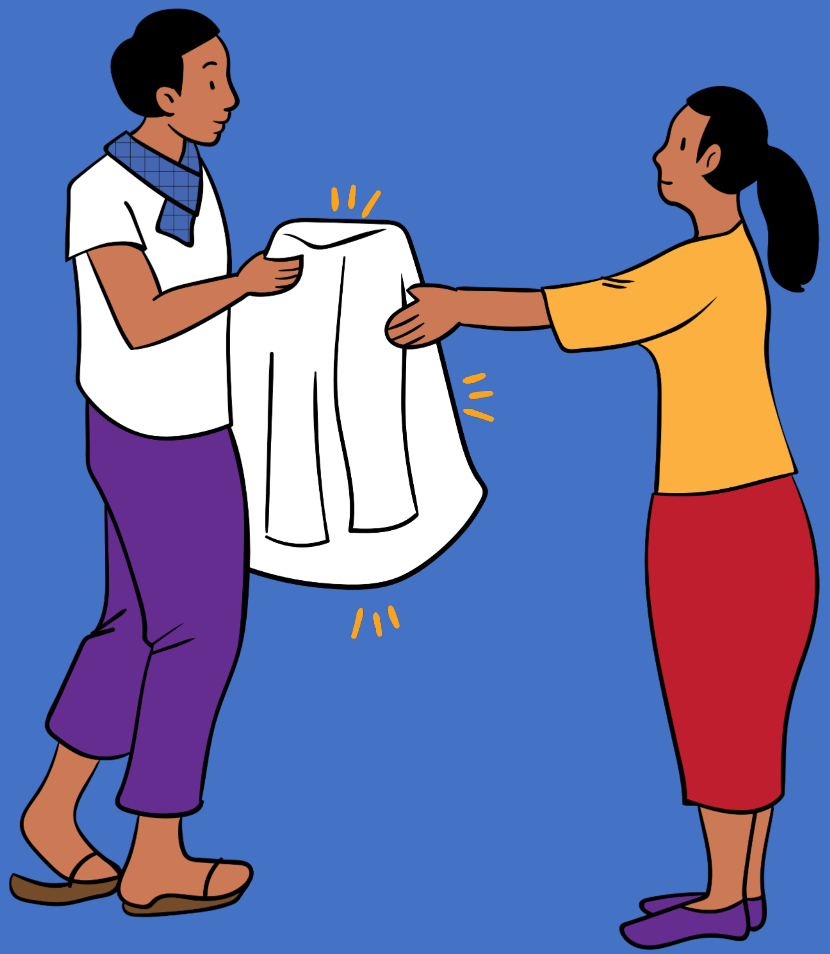

FALSE!

The truth is that your treated clothing is only for you to use. You need it to protect yourself from mosquitoes, and only you have had the proper training to understand how to use them properly and how to be safe when using them.

Truth or Myth?

It's important to keep repellent sheets dry.

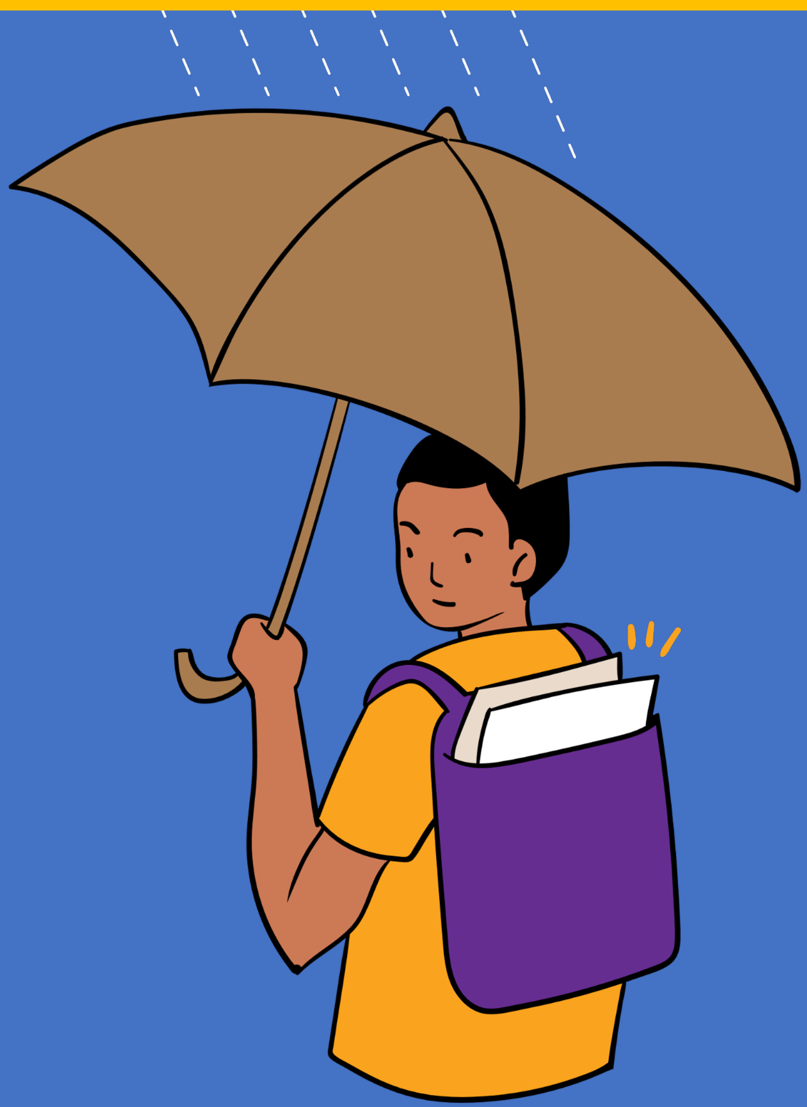

TRUTH!

The repellent sheets are the most powerful when you keep them out of the rain. You can hang them indoors or inside a shelter in the farm or forest where you spend time. Keep them dry so they can keep you protected from mosquito bites!

What questions do you hear about the products? Do you have any questions you'd like us to try to answer today or in our next visit?

Wrap Up and P.L.A.N.

I'm so glad we had a chance to talk about your experience using these new products. You're so much better protected today than you were before! Going forward, **don't forget to P.L.A.N**:

Prepare to carry and use all prevention products every day, even when you go to the forest

Leave all worries behind, knowing that you have full protection from mosquito bites

Always encourage others in your family or ranger group to use all 3 products, but

Never share the products outside your family or ranger group

I'll see you soon and when I do I hope you'll be able to tell me about how these products are freeing you from unnecessary worry about illnesses and hassle caused by mosquito bites.
