# Supplementary material for: Evaluating the acceptability and feasibility of new mosquito bite prevention tools in a “forest pack” to support malaria elimination in Cambodia
Source: Malar J. 2025 Nov 27;24:443. doi: 10.1186/s12936-025-05682-2 (PMC12715958; doi:10.1186/s12936-025-05682-2)
Supplement: Supplementary file 6 — Additional file6 (PDF 5474 KB) [file 12936_2025_5682_MOESM6_ESM.pdf]

វិធីការពារខ្លួនយ៉ាងពេញលេញពីមូសខាំ

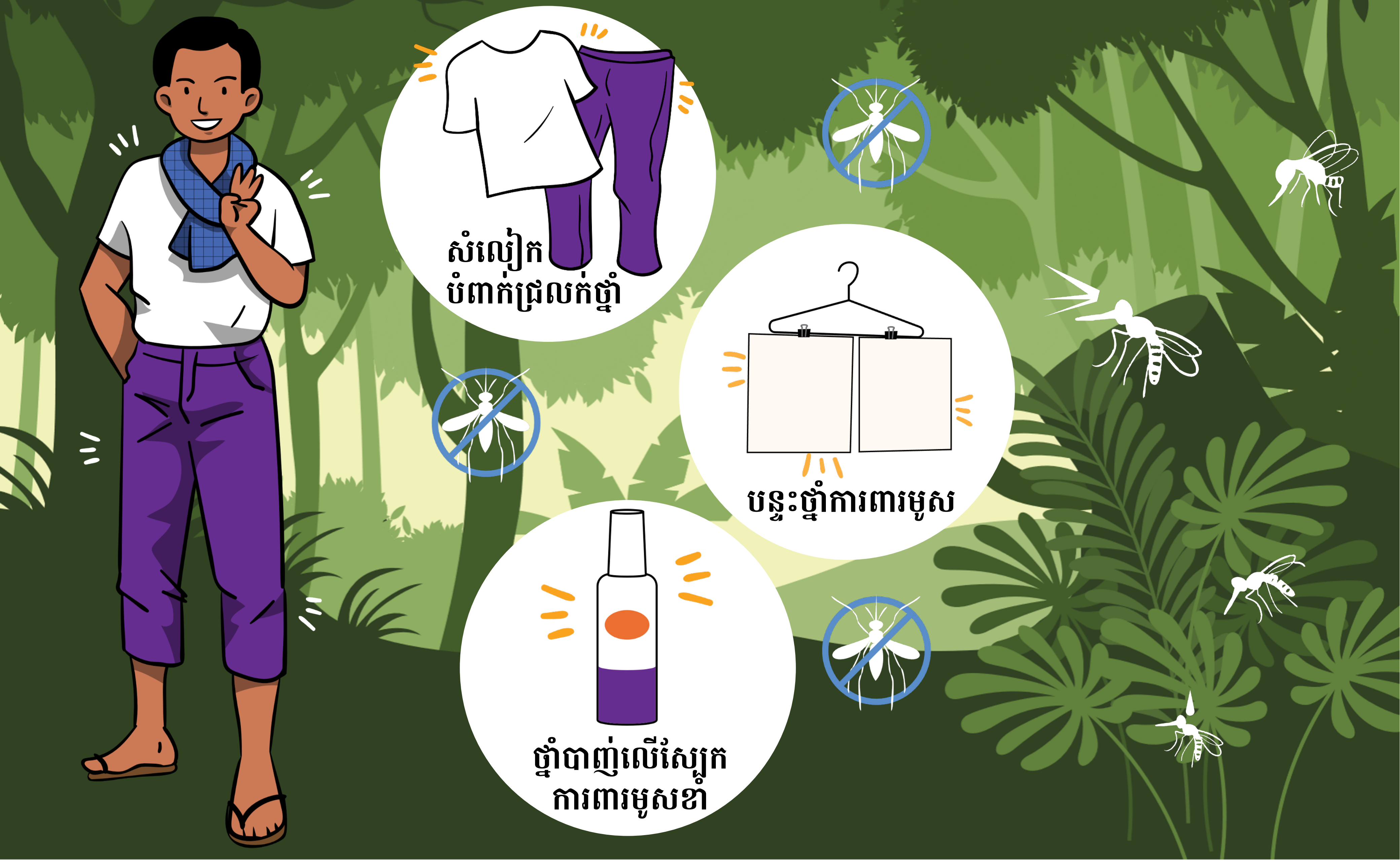

ចំណុចពិភាក្សាសម្រាប់អ្នកស្ម័គ្រចិត្តព្យាបាលគ្រុនចាញ់ភូមិ អ្នកស្ម័គ្រចិត្តព្យាបាលគ្រុនចាញ់ចល័ត ប្រធានភូមិ និងប្រធានសហគមន៍៖

ជំរាបសួរ ហើយតើអ្នកសុខសប្បាយជាទេតាំងពីយើងជួបគ្នាលើកមុន ?

ខ្ញុំនៅទីនេះដើម្បីផ្តល់នូវផលិតផលថ្មី និងដើម្បីរំលឹកអ្នកអំពី
អត្ថប្រយោជន៍នៃការប្រើប្រាស់ផលិតផលថ្មីទាំង ៣ នេះ។

ជីវិតតែងមានភាពមិនច្បាស់លាស់ និងមានហានិភ័យ។ រឿងល្អនោះគឺថា បានប្រើប្រាស់ផលិតផលថ្មីទាំង ៣នេះ នឹងអាចកាត់បន្ថយហានិភ័យមួយក្នុងចំណោមហានិភ័យទាំងនោះ និងការពារខ្លួនអ្នកយ៉ាងពេញលេញពីមូសខាំ និងជំងឺផ្សេងទៀតដែលពាក់ព័ន្ធនឹងមូសខាំ។

ហានិភ័យតិច មានន័យថាអ្នក និងគ្រួសាររបស់អ្នកមានការរីកចម្រើន។
តោះមកនិយាយបន្ថែមពីវិធីប្រើផលិតផលទាំង ៣ ដើម្បីអោយអ្នកកាន់តែមានថាមពលខ្លាំងជៀសវាងពីមូសខាំ!

រូបភាពទាំងពីរនេះបង្ហាញពីកន្លែងដែលត្រូវ
ប្រើសម្លៀកបំពាក់ជ្រលក់ថ្នាំ

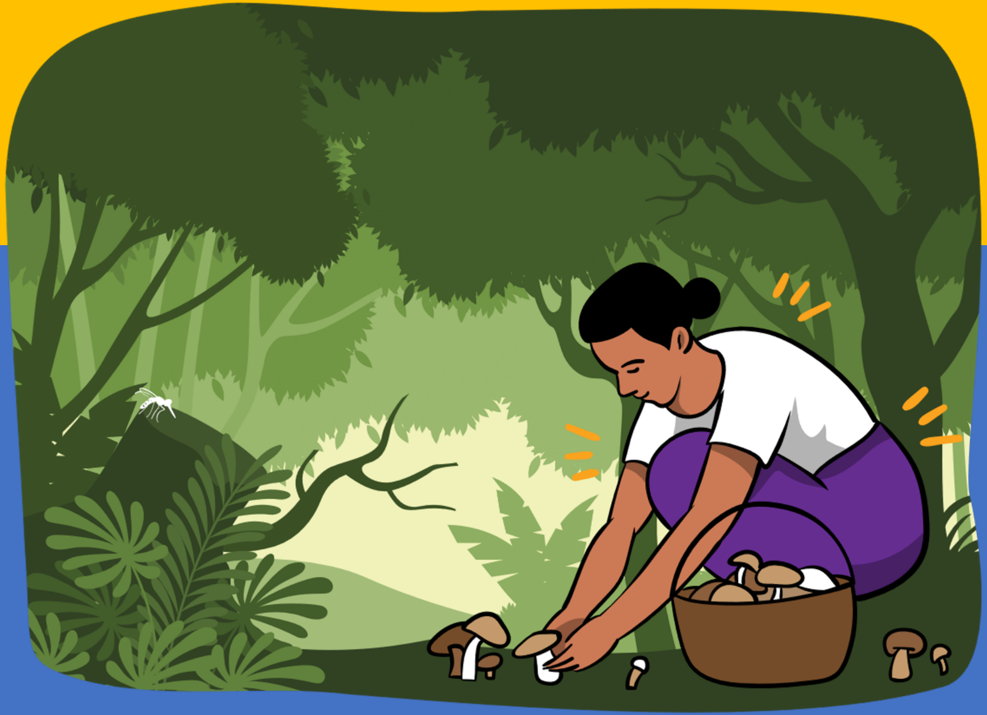

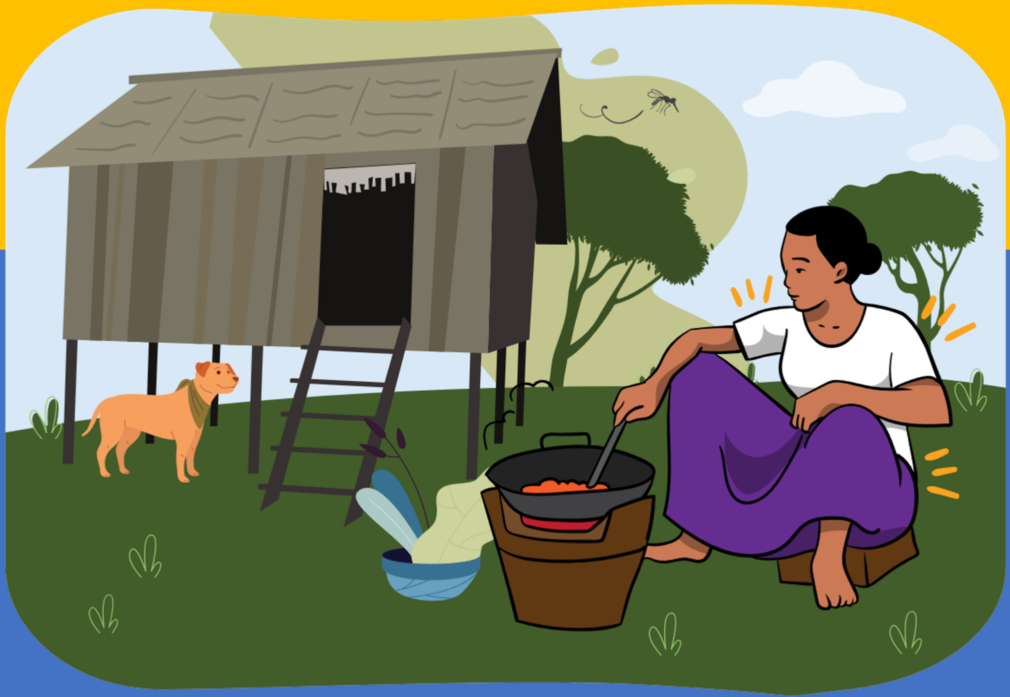

មធ្យោបាយដ៏ល្អបំផុតក្នុងការប្រើសម្លៀកបំពាក់ជ្រលក់ថ្នាំគឺត្រូវស្លៀកសម្លៀកបំពាក់នេះឱ្យបានច្រើនតាមដែលអាចធ្វើទៅបាន ជាពិសេសពេលចេញទៅក្រៅ ដូចជាពេលអ្នកចូលព្រៃ និងពេលអ្នកនៅជិតផ្ទះរបស់អ្នក។

រូបភាពខាងលើទាំងពីរនេះបង្ហាញពីទីតាំងដែលអ្នកអាចប្រើសម្លៀកបំពាក់ជ្រលក់ថ្នាំរបស់អ្នក ដូច្នេះវាអាចឱ្យអ្នកបានទទួលការការពារនៅកន្លែងទាំងនោះដែលជាកន្លែងប្រឈមនឹងការខាំពីមូស។

អ្នកខ្លះយល់ច្រឡំថា សម្លៀកបំពាក់ជ្រលក់ថ្នាំប្រើតែពេលទៅព្រៃ **ប៉ុន្តែវិធីការពារល្អបំផុតគឺត្រូវស្លៀកសម្លៀកបំពាក់នេះគ្រប់ពេលដែលអ្នកនៅខាងក្រៅផ្ទះ។**

ចូរគិតថាវាជាឈុតដ៏អស្ចារ្យរបស់អ្នក!

ប្រសិនបើសម្លៀកបំពាក់ មានក្លិនដែលជាក្លិនទើបជ្រលក់ថ្នាំថ្មីៗ ហើយ ក្លិននោះរំខានអ្នក សូមបោកសម្លៀកបំពាក់នោះម្តង ឬពីរដងដើម្បីបំបាត់ក្លិន។ សម្លៀកបំពាកអាចមានប្រសិទ្ធភាព និងអាចបោកគក់ដល់ទៅ ២៥ ដង!

តើអ្នកមានសំនួរអ្វីដែរឬទេ?

តើរូបភាពមួយណាក្នុងចំណោមរូបភាពទាំងពីរនេះ បង្ហាញពីវិធីល្អបំផុតក្នុងការប្រើបន្ទះថ្នាំការពារមូស ?

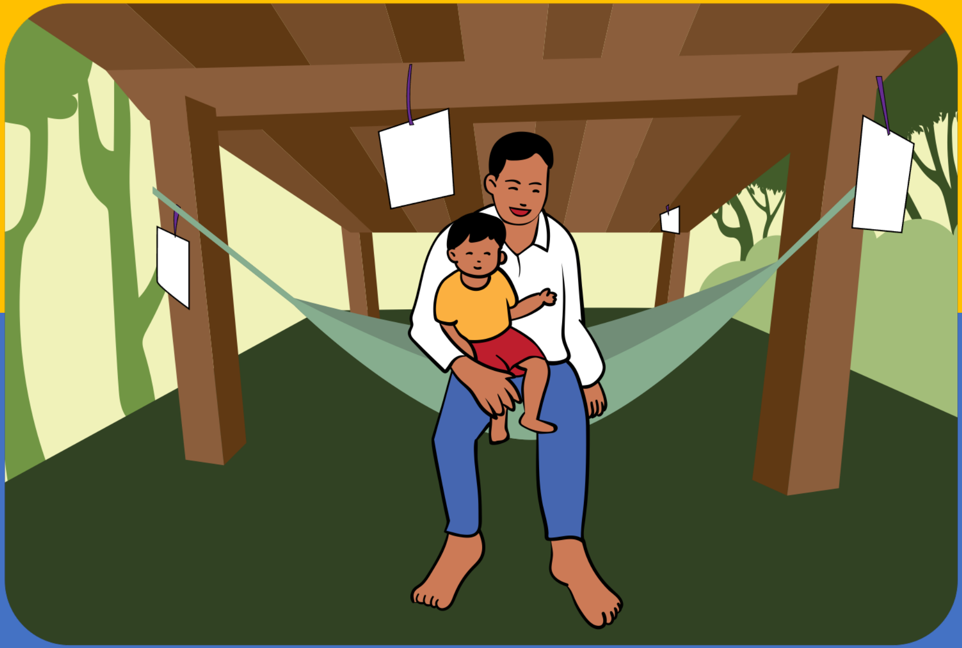

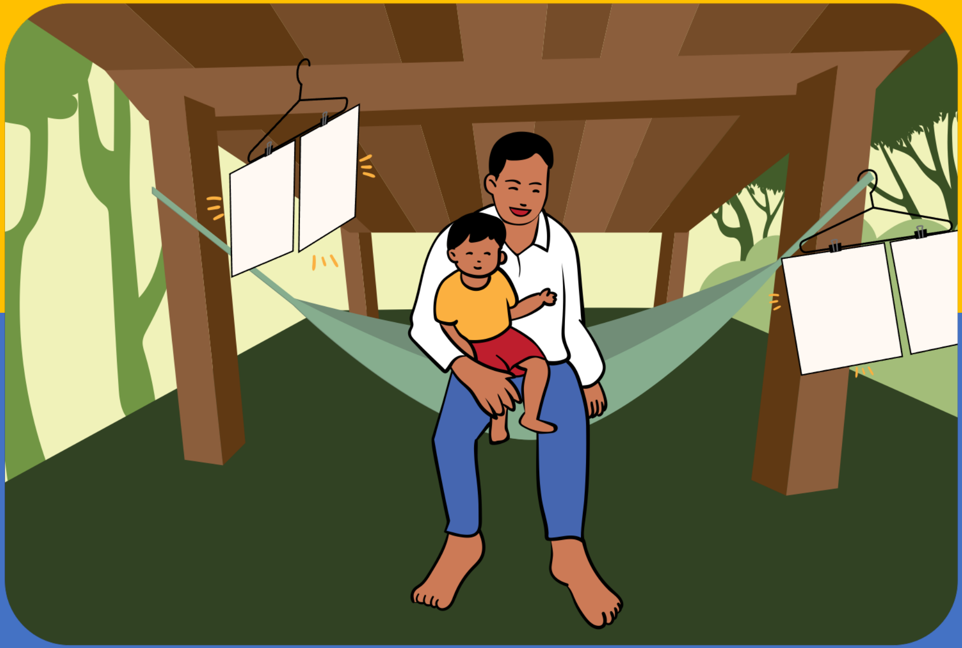

មធ្យោបាយដ៏ល្អបំផុតក្នុងការប្រើសប្រាស់បន្ទះថ្នាំការពារមូសគឺត្រូវព្យួរបន្ទះនេះជាគូ (២បន្ទះ!)

រូបភាពនៅខាងស្តាំដែលបង្ហាញពីវិធីព្យួរវាដ៏ត្រឹមត្រូវ ពោលគឺ២បន្ទះជាប់គ្នា (ជំនួសឱ្យតែមួយសន្លឹក)។
ព្យួរវានៅកន្លែងណាដែលអ្នកចំណាយពេលច្រើន និងយកវាទៅព្រៃជាមួយអ្នក។

ព្យួរបន្ទះនេះយ៉ាងហោចណាស់២ម៉ែត្រពីដីហើយកុំឱ្យក្មេងកាន់លេង។ បន្ទះនេះមិនមានក្លិនទេ ប៉ុន្តែមានសារធាតុថ្នាំការពារមូសនៅខាងក្នុងរួចហើយ មិនចាំបាច់បាញ់ថ្នាំការពារមូសលើវាទៀតឡើយ!

ត្រូវតែពាក់ស្រោមដៃជានិច្ចនៅពេលអ្នកប៉ះបន្ទះថ្នាំការពារមូសនេះ!

តើអ្នកមានបញ្ហាក្នុងការប្រើបន្ទះនេះដូចដែលបានណែនាំខាងលើនេះទេ?

តើរូបភាពខាងក្រោមមួយណាដែលបង្ហាញពីវិធីល្អ បំផុតក្នុងការប្រើថ្នាំការពារលើស្បែក ?

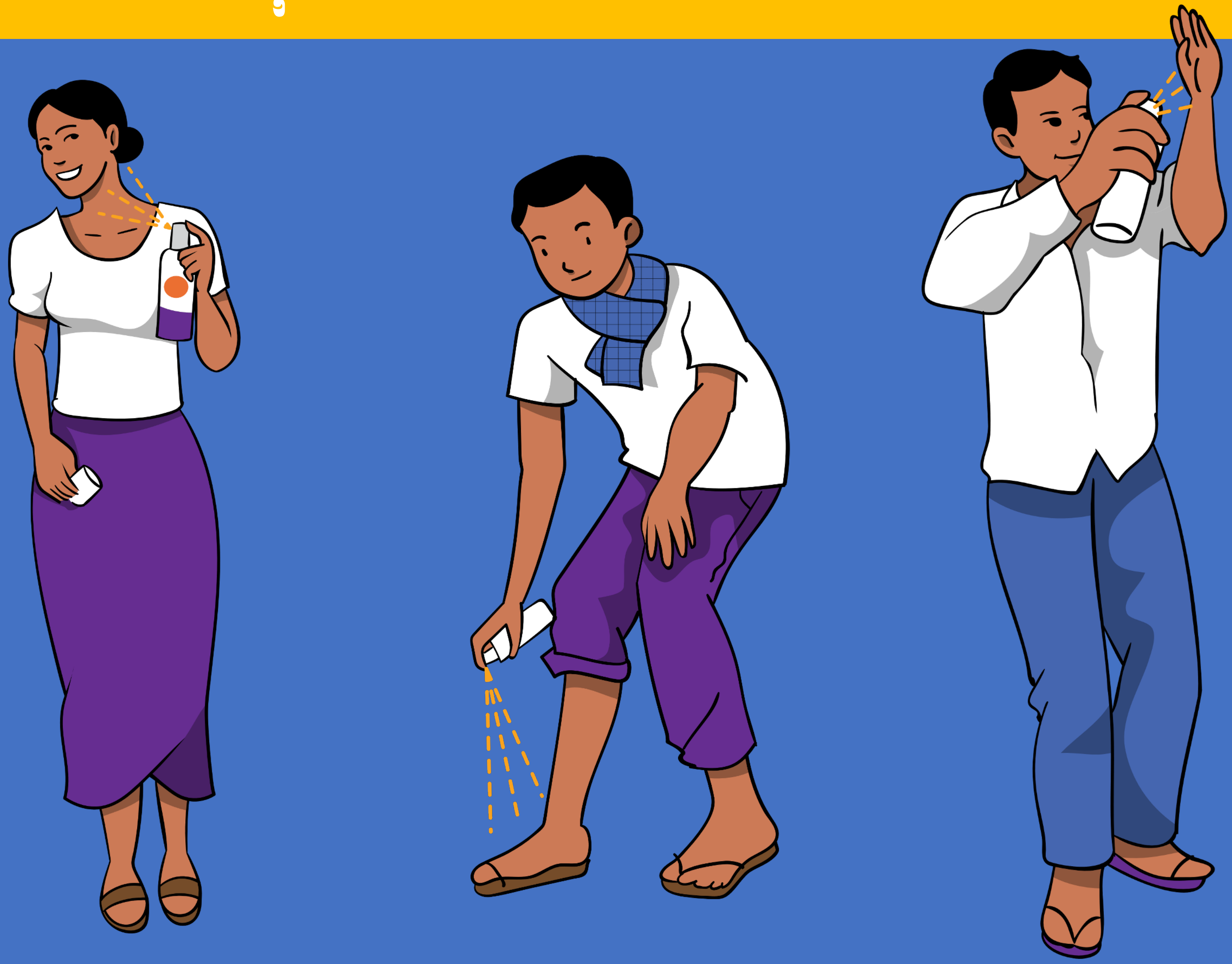

មធ្យោបាយដ៏ល្អបំផុតក្នុងការប្រើថ្នាំបាញ់លើស្បែកគឺបាញ់ត្រង់ផ្នែកនៃរាងកាយណាដែលអាចប្រឈមនឹងមូសខាំ។

រូបភាពទាំងអស់នេះមានសារៈសំខាន់ទាំងបុរស និងស្ត្រីដើម្បីប្រើប្រាស់ថ្នាំការពារមូសខាំលើស្បែក**ជារៀងរាល់ថ្ងៃ** ដើម្បីការពារផ្នែកនៃរាងកាយរបស់អ្នកអោយបានពេញលេញ។

ទោះបីជាអ្នកពាក់អាវដៃវែងឬខោជើងវែងក៏ដោយ អ្នកគួរតែបាញ់ដៃ និងជើងរបស់អ្នកត្រង់ផ្នែកខាងក្រោមសម្លៀកបំពាក់របស់អ្នក ដូច្នេះវាអាចការពារអ្នកបានល្អ**ទោះបីអ្នកនៅផ្ទះក៏ដោយ។**

ចូរចងចាំថា បាញ់ថ្នាំនេះលើស្បែកតែប៉ុណ្ណោះ មិនអាចបាញ់លើមុង មុង អង្រឹង ភួយ ខ្នើយ ឬនៅលើសន្លឹកថ្នាំការពារមូសដែលបានព្យាបាលនោះទេ។

តើអ្នកមានចម្ងល់អំពីថ្នាំបាញ់លើស្បែកការពារមូសខាំដែរឬទេ ?

តើផលិតផលមួយណាដែលអ្នកគិតថាសំខាន់ក្នុងការ
ប្រើប្រាស់ ប្រសិនបើអ្នកមានមុខ ឬមុខអទ្រើខជ្រលក់ថ្នាំ
រួចហើយ ?

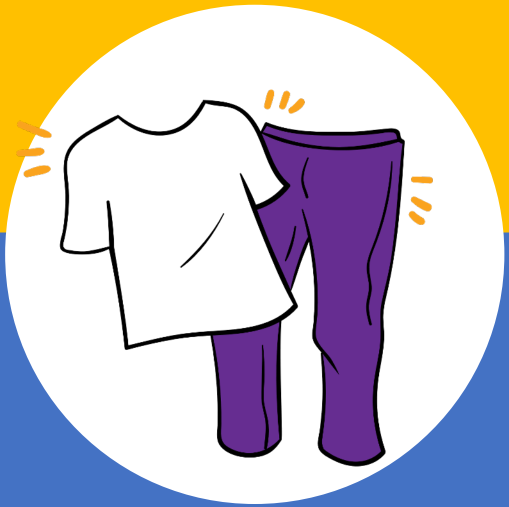

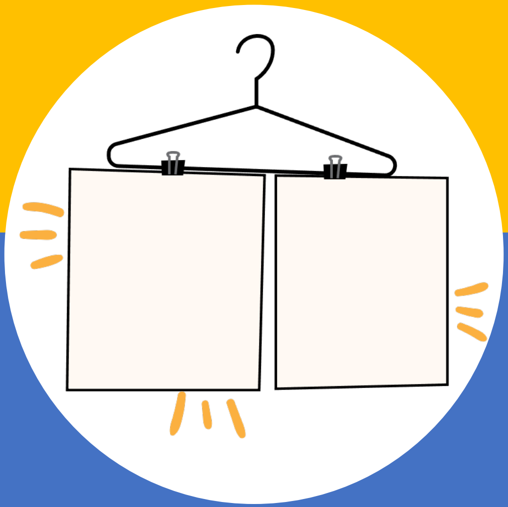

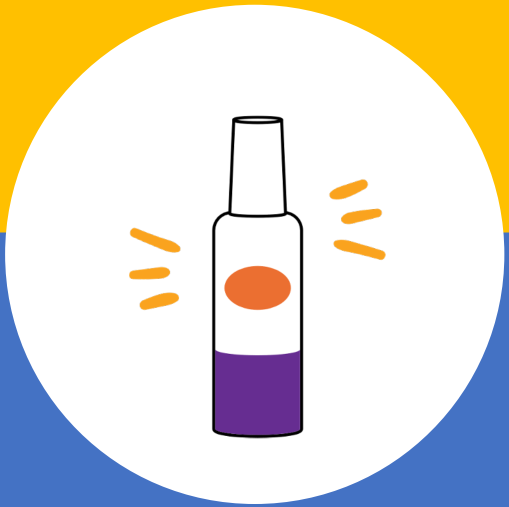

តាមពិតទៅវិធីល្អបំផុតសម្រាប់ការការពារមូសខាំ និងជំងឺដ៏ទៃទៀតដែលពាក់ព័ន្ធនឹងមូសគឺត្រូវប្រើប្រាស់ផលិតផលទាំងបីរួមជាមួយនឹងមុងជ្រលក់ថ្នាំ! វាដូចជាអ្នកដាំដំណាំដោយមិនអាចគ្មានទឹក និងជីអញ្ចឹង រួចហើយអ្នកត្រូវមានពូជដំណាំដ៏ល្អផងដែរ។ ហេតុអ្វីបានជាអ្នកមិនប្រើប្រាស់ផលិតផលការពារពេញលេញពីមូសខាំ?

តើអ្នកបានសុំអោយមនុស្សដែលស្នាក់នៅជាមួយអ្នក ឬធ្វើការជាមួយអ្នកឱ្យជួយរំលឹកអ្នកឱ្យយក និងប្រើប្រាស់ផលិតផលទាំង ៣នេះជាមួយអ្នកនៅពេលដែលអ្នកចេញក្រៅផ្ទះដែរឬទេ?

ផ្ទះផ្សេងទៀតដែលខ្ញុំទៅលេងប្រាប់ខ្ញុំថា នៅពេលដែលពួកគេជួយណែនាំគ្នា ពួកគេបានជៀសផុតពីមូសខាំបានច្រើន ដោយប្រើប្រាស់ផលិតផលទាំង ៣ នេះជារៀងរាល់ថ្ងៃ។

តើអ្នកធ្វើដូចម្តេចដើម្បីចងចាំប្រើផលិតផលទាំង ៣នេះជារៀងរាល់ថ្ងៃចាប់តាំងពីពេលនេះរហូតដល់ពេលដែលខ្ញុំជួបអ្នកនៅខែក្រោយ?

ប្រជាជន និងមន្ត្រីអនុរក្សព្រៃក្តីដែលបានជួយរំលឹកគ្នាប្រើប្រាស់ផលិតផលនេះក្នុងបរិមាណអតិបរមា(បានច្រើន) នឹងមានថាមពលការពារមូសបន្ថែម។

ពិត ឬមិនពិត?

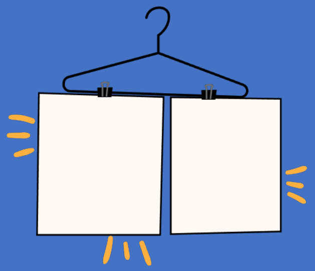

សន្លឹកថ្នាំការពារមូស
មានសុវត្ថិភាពក្នុងការ
ប្រើប្រាស់សម្រាប់
មនុស្សគ្រប់វ័យ

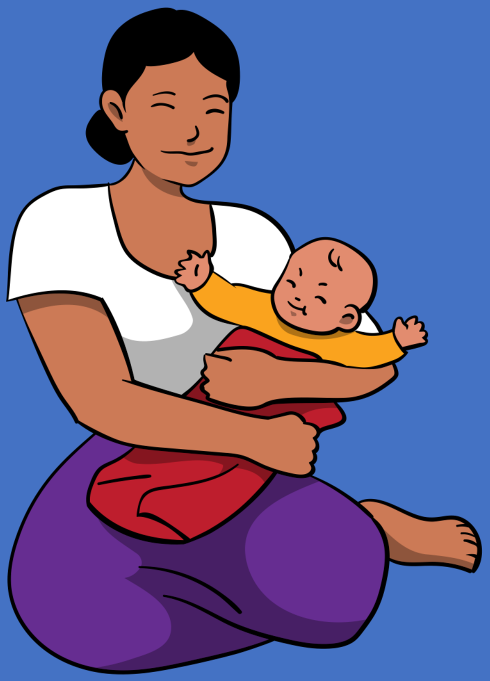

ការពិត!

សន្លឹកថ្នាំការពារមូសគឺមានសុវត្ថិភាពខ្លាំងណាស់សំរាប់ប្រើប្រាស់ជុំវិញអ្នក។ ពួកវាមិនមានក្លិនទេ ហើយមិនធ្វើឱ្យអ្នកមានអារម្មណ៍ចង់ក្អួតនោះឡើយ។ សូមចងចាំថាអ្នកត្រូវប្រើស្រោមដៃនៅពេលអ្នកប៉ះសន្លឹកនេះ។

ពិត ឬមិនពិត?

មូសខាំគឺរំខាន ប៉ុន្តែវា
មិនអាចបង្កជាជំងឺ
ធ្ងន់ធ្ងរបានទេ។

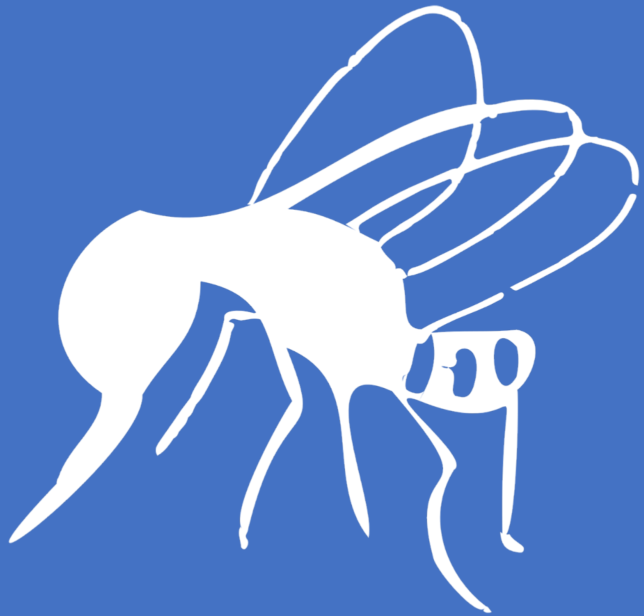

មិនពិត!

តាមពិតទៅ មូសអាចចម្លងជំងឺគ្រោះថ្នាក់ដូចជាជំងឺគ្រុនចាញ់ និងគ្រុនឈាម។ អ្នកកាន់តែប្រើផលិតផលការពារខ្លួនអ្នកពីមូសខាំបានកាន់តែច្រើន អ្នកនឹងមានសុវត្ថិភាពពីជំងឺទាំងនេះ។

ពិត ឬមិនពិត ?

គ្រួសារនីមួយៗត្រូវការសម្លៀកបំពាក់ជ្រលក់ថ្នាំការពារ
មូសខាំតែមួយប៉ុណ្ណោះ ?

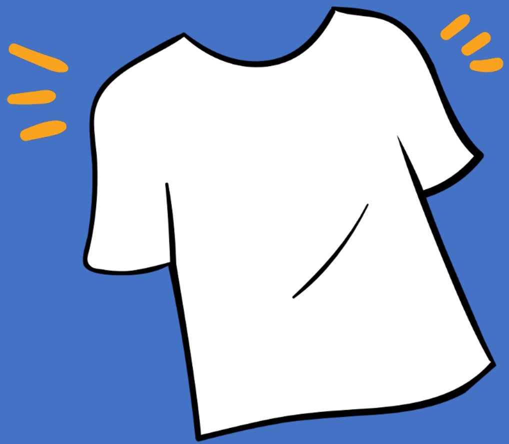

VS

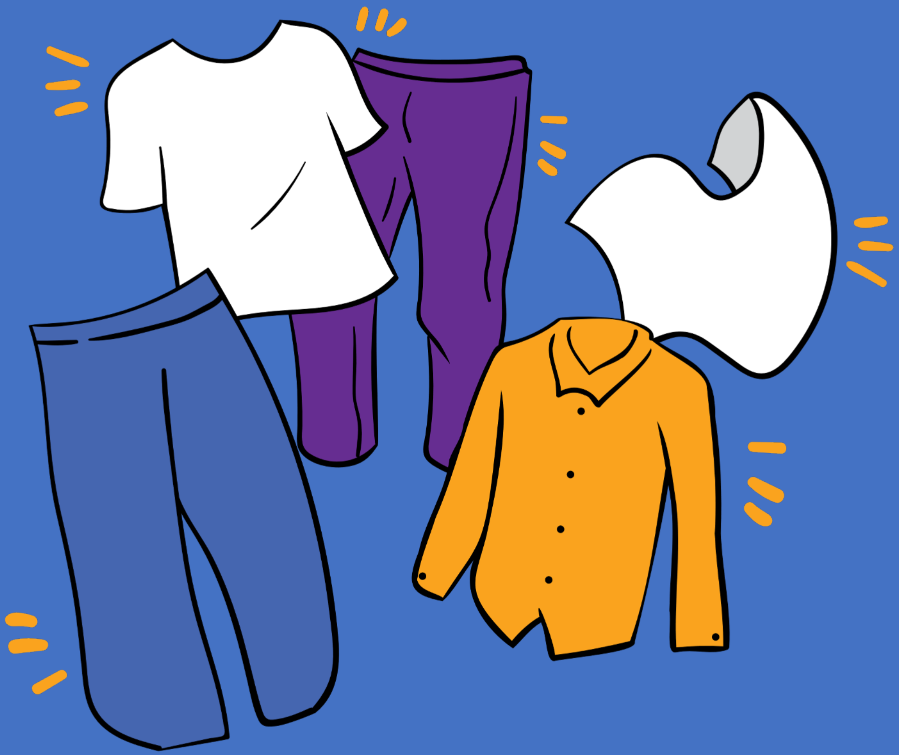

មិនពិត!

តាមពិតទៅមនុស្សម្នាក់ៗគួរតែទទួលបានសម្លៀកបំពាក់ជ្រលក់ថ្នាំការពារមូសខាំចាប់ពី ៤ ទៅ ៥ ខោឬអាវ ដូច្នេះអ្នកអាចស្លៀកសម្លៀកបំពាក់ជ្រលក់ថ្នាំផ្សេងគ្នាជារៀងរាល់ថ្ងៃបាន។

ត្រូវ ឬមិនត្រូវ?

វាមិនអីទេបើអោយសម្លៀកបំពាក់
ជ្រទ្បក់ថ្នាំការពារមូសរបស់ខ្ញុំទៅ
បងប្អូនរបស់ខ្ញុំនៅក្នុងភូមិជិតគ្នា
ប្រសិនបើគាត់ទៅចំការហើយគាត់
ត្រូវការការការពារបន្ថែមពីមូស

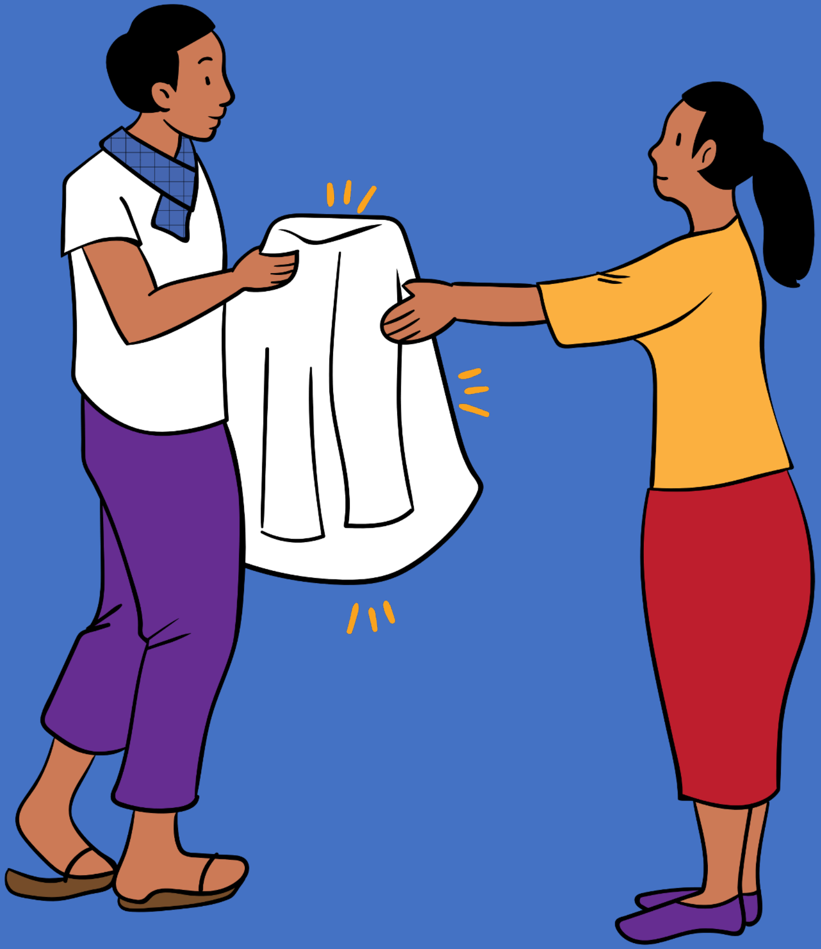

មិនត្រឹមត្រូវ!

តាមពិតទៅសម្លៀកបំពាក់ជ្រលក់ថ្នាំការពារមូសរបស់
អ្នកគឺសម្រាប់តែអ្នកប្រើប៉ុណ្ណោះ។ អ្នកត្រូវការវាដើម្បី
ការពារខ្លួនអ្នកពីមូស ហើយមានតែអ្នកប៉ុណ្ណោះដែល
បានទទួលការបណ្តុះបណ្តាលត្រឹមត្រូវនិងយល់ពី
របៀបប្រើប្រាស់វាឱ្យបានត្រឹមត្រូវ និងមានសុវត្ថិភាព
នៅពេលប្រើវា។

ត្រូវ ឬខុស ?

វាសំខាន់ណាស់ក្នុងការ
រក្សាសន្លឹកថ្នាំការពារ
មូសខាំឱ្យស្ងួត។

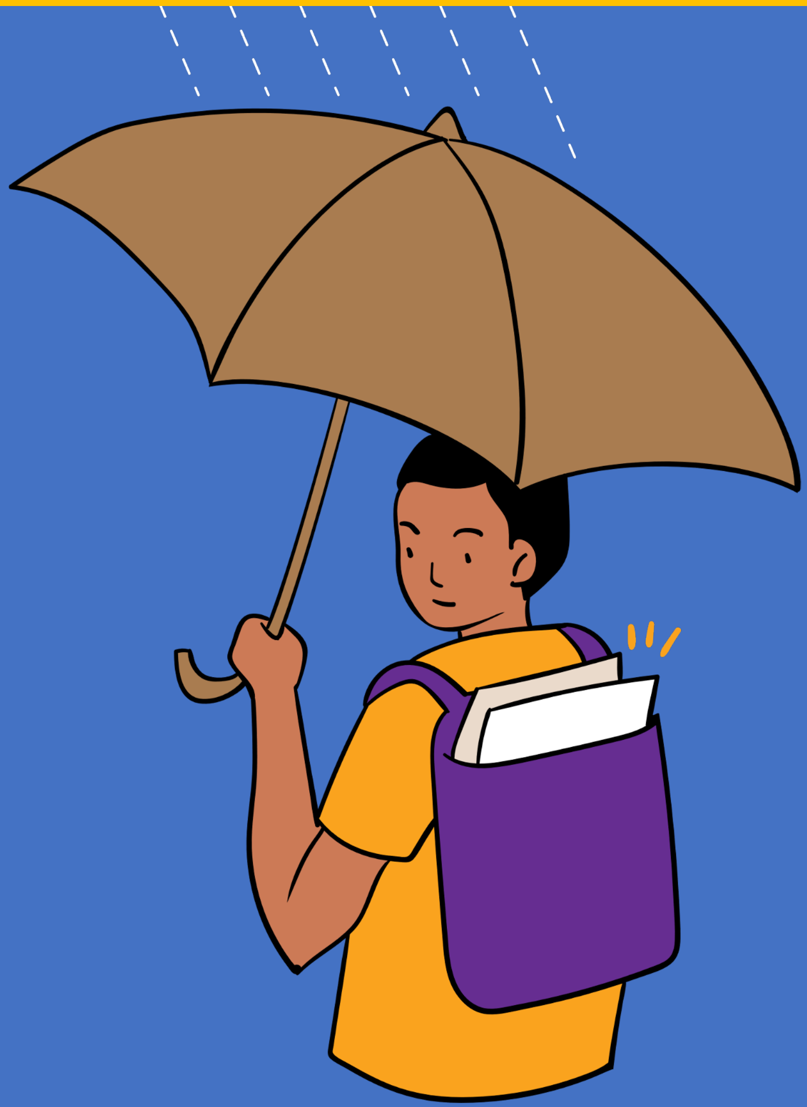

ត្រូវ!

សន្លឹកថ្នាំការពារមូសមានប្រសិទ្ធិភាពខ្ពស់ដ៏រាបណារក្សាវាឱ្យផុតតំណក់ភ្លៀង។ អ្នកអាចព្យួរវាក្នុងផ្ទះ ឬក្នុងទីជម្រកឯចំការ ឬព្រៃដែលអ្នកបានទៅ។ ទុកវាឱ្យស្ងួត ទើបវាអាចការពារអ្នកពីមូសខាំ!

តើអ្នកឮសំណួរអ្វីខ្លះអំពីផលិតផលទាំងនេះ?

តើអ្នកមានសំណួរណាមួយដែលអ្នកចង់ឱ្យយើងឆ្លើយនៅថ្ងៃនេះ ឬនៅពេលជួបគ្នាលើកក្រោយដែរឬទេ?

រំលឹក និងដាក់ផែនការ (P.L.A.N)

ខ្ញុំរីករាយណាស់ដែលយើងមានឱកាសនិយាយអំពីបទពិសោធន៍របស់អ្នកក្នុងការប្រើផលិតផលការពារមូសខាំថ្មីទាំងនេះ។ ថ្ងៃនេះអ្នកបានការពារមូសបានប្រសើរជាងមុន!
ទៅមុខ**កុំភ្លេចរៀបចំផែនការ** (**P.L.A.N**)

P: **ត្រៀមខ្លួន៖** ត្រៀមខ្លួននឹងប្រើប្រាស់គ្រប់ផលិតផលការពារមូសខាំជារៀងរាល់ថ្ងៃសូម្បីចូលព្រៃក៏ដោយ
L: **បោះចោល៖** បោះចោលភាពកង្វល់ទាំងឡាយដោយដឹងថាអ្នកមានការការពារពេញលេញពីមូសខាំ

A: **តែងតែ៖** តែងតែលើកទឹកចិត្តសមាជិកក្នុងគ្រួសារ ឬក្រុមអភិរក្សព្រៃរបស់អ្នកឱ្យប្រើផលិតផលការពារមូសទាំង៣ ក៏ប៉ុន្តែ

N: **ហាម ឬកុំ៖** កុំចែករំលែកផលិតផលការពារមូសខាំទាំងនេះទៅអ្នកផ្សេង

ខ្ញុំនឹងជួបអ្នកឆាប់ៗនេះ ហើយពេលនោះខ្ញុំសង្ឃឹមថាអ្នកនឹងអាចប្រាប់ខ្ញុំពីផលិតផលទាំងនេះដែលអាចធ្វើអោយអ្នកឈប់ព្រួយបារម្ភពីជំងឺបណ្តាលពីមូសខាំ និងការរំខាននានាដែលបណ្តាលមកពីមូសខាំផងដែរ។
