# Supplementary material for: Evaluating the acceptability and feasibility of new mosquito bite prevention tools in a “forest pack” to support malaria elimination in Cambodia
Source: Malar J. 2025 Nov 27;24:443. doi: 10.1186/s12936-025-05682-2 (PMC12715958; doi:10.1186/s12936-025-05682-2)
Supplement: Supplementary file 7 — Additional file7 (PDF 426 KB) [file 12936_2025_5682_MOESM7_ESM.pdf]

REMEMBER to use *all* 3 products *every day and night* for the best protection from mosquito bites!

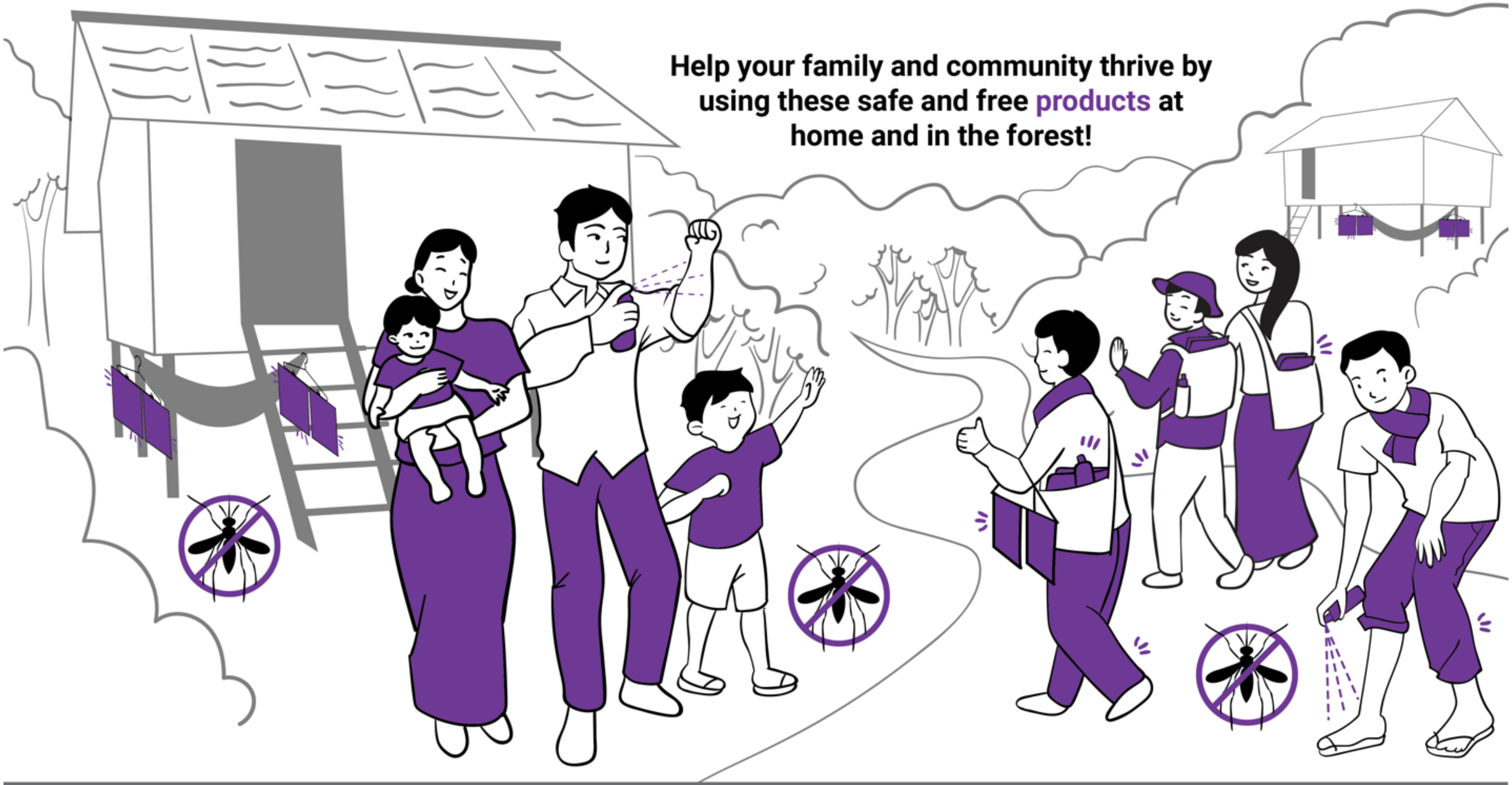

Questions? Ask your Village Malaria Worker or Village Leader for Assistance!
