# Supplementary material for: Evaluating the acceptability and feasibility of new mosquito bite prevention tools in a “forest pack” to support malaria elimination in Cambodia
Source: Malar J. 2025 Nov 27;24:443. doi: 10.1186/s12936-025-05682-2 (PMC12715958; doi:10.1186/s12936-025-05682-2)
Supplement: Supplementary file 8 — Additional file8 (PDF 128 KB) [file 12936_2025_5682_MOESM8_ESM.pdf]

កុំភ្លេចប្រើផលិតផលទាំង ៣ នេះ ជារៀងរាល់ថ្ងៃ និងយប់ ដើម្បីការការពារដ៏ល្អបំផុត ពីមូសខាំ!

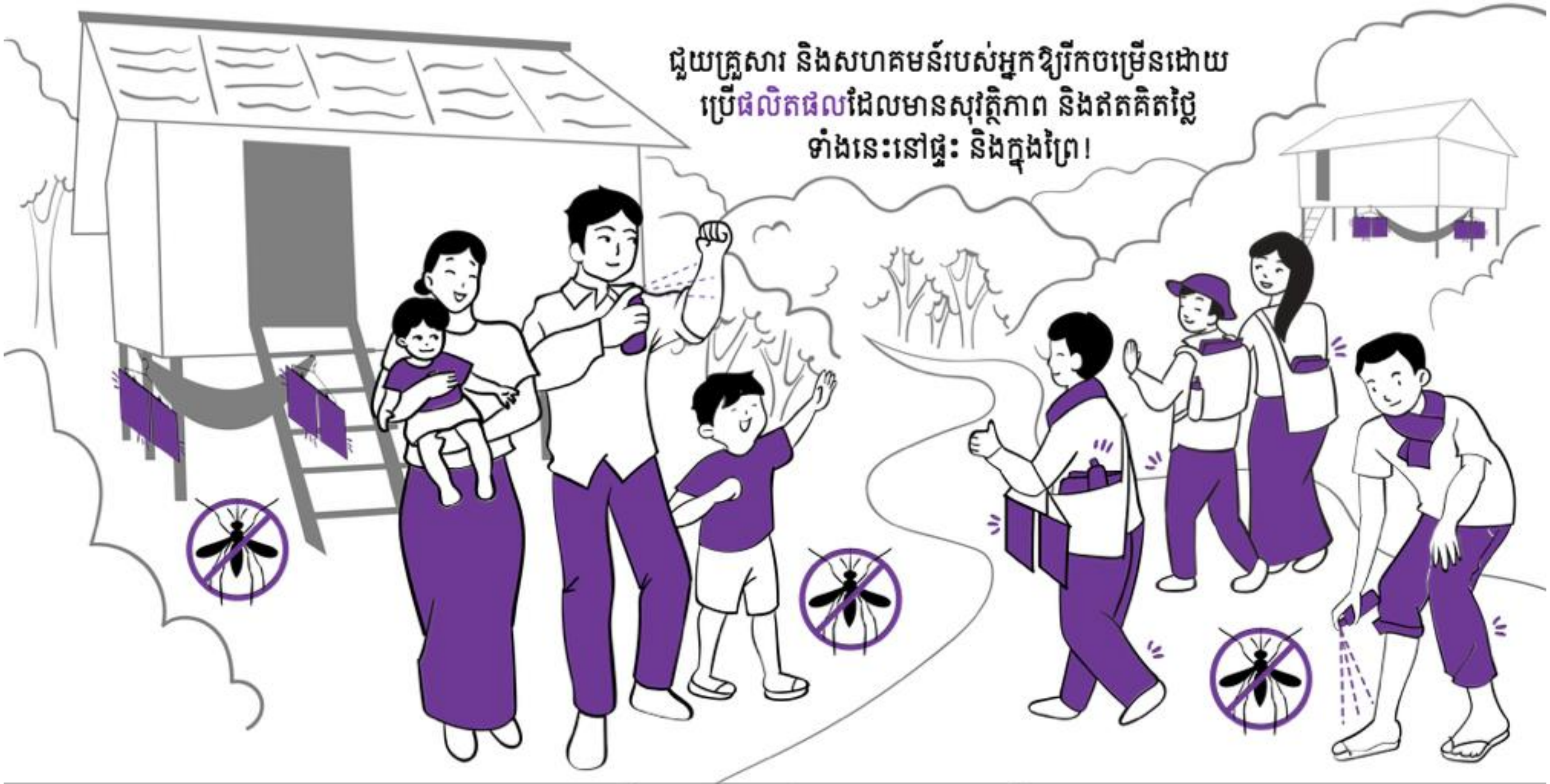

មានសំណួរ ឬ ចម្ងល់? សូមស្វែងរកជំនួយពីមេភូមិ ឬអ្នកស្ម័គ្រចិត្តភូមិព្យាបាលជំងឺគ្រុនចាញ់នៅក្នុងភូមិរបស់អ្នក!
