# Supplementary material for: Evaluating the acceptability and feasibility of new mosquito bite prevention tools in a “forest pack” to support malaria elimination in Cambodia
Source: Malar J. 2025 Nov 27;24:443. doi: 10.1186/s12936-025-05682-2 (PMC12715958; doi:10.1186/s12936-025-05682-2)
Supplement: Supplementary file 9 — Additional file9 (PDF 174 KB) [file 12936_2025_5682_MOESM9_ESM.pdf]

Appendix 2 – Key Informant Interview and Focus Group Discussion Guide (End User)

BITE Project: Qualitative interview guide

**ID code** [1]	:
**Village name**	:
**Date of interview**	:
**GPS Coordinate**	:
**Group**	:
**Ethnic group identify**[2]	:
**Gender**	:
**Age**	:

**COM-B**[3]	**TDF**	**Question**
Psychological capability	Knowledge	What is your understanding of malaria?<br><br>• Prompt: How is it transmitted, what are the signs and symptoms, how is it treated)<br><br>What products are available in your community to protect people from mosquito bite?<br><br>• Prompt: How do you use each of these products?

[1] This should be the same ID code as used for the rest of the DBS collection. All participants will be individuals who have taken part in all three cross-sectional surveys, and we can use the same ID.

[2] May need a translator if the participant is indigenous group

[3] **COM-B : Capability, Opportunity and Motivation**

Psychological capability	Memory, attention and decision processes	To what extent do you feel you need to protect yourself and your family from mosquito bites?<br><br>Why?<br><br>Why not?<br><br>• Prompt: What do you think is/are the most effective method(s) for protecting yourself or family members from mosquito bites?<br><br>Why?
Physical capability	Skills	To what extent are you confident in your ability to protect yourself and your family member from mosquito bites?<br><br>• Prompt: In the last 1 month, have you used the following products that were given to you: Spatial repellent (PIRK, treated sheet), treated clothing (Etofenprox, ETO), or Tropical repellent (spray, personal repellent))?<br><br>• Prompt: Who did you receive from (VMW/MMW/VC)?<br><br>• Prompt: Which of the three products did you use the most and why?<br><br>• Prompt: why did you use the other product/s less?
Social opportunity	Social influences	To what extent did support from your family or friends help influence your use of this product (XXXX)? (Ask for each product they received)<br><br>How did they support?<br><br>• Prompt: What could your family, friends or others (including the VMW/MMW/VC) in the community do to motivate people like you to use this product (XXXX) consistently to protect from mosquito bite?<br><br>Probe: whose support would influence you the most and why?
Physical opportunity	Environmental context and resources	Does anything in the location where you live and work help or hinder you in using this product (XXXX)?<br><br>• Prompt: physical, environmental factors such as: “lack of easy places to hang treated sheets in areas of the forest where I sleep”, seasonality (rainy, windy, dry season, etc.)

Reflective motivation	Belief about capabilities	On a scale of 1 to 5 where 1 is very easy and 5 is very difficult, how difficult/easy do you think it is for someone like you to use this product (XXXX) that protect them and their family members from mosquito bites?<br><br>• Prompt: Why (for each product)?<br><br>What is difficult about using (for products that scored 4 or 5)?<br><br>What is easy about using (for products that scored 1 or 2)?
Reflective motivation	Intention	To what extent would you intend to continue using this product (XXXX) to protect from mosquito bites in future, if it were available to you?<br><br>• Prompt: If not, why not?
Reflective motivation	Goals	If this product were available in your nearest market, to what extent would you be likely to purchase this product after the BITE Project ends (XXXX).
Reflective motivation	Belief about consequences	What do you think are the health implications of using this product (XXXX) to protect from mosquito bite?<br><br>• Prompt: Any negative consequences and what are the benefits?
Automatic motivation	Emotion	How did using this product (XXXX) to protect from mosquito bites make you feel?<br><br>• Prompt: Can you give me 2 words to describe how you feel about each of the 3 products?<br><br>Let's start with treated clothing (Etofenprox, ETO).<br>What about topical repellent (spray, personal repellent)?<br>What about spatial repellent (PIRK, treated sheets)?<br>• Prompt: To what extent would you recommend this product to other forest goers?<br>• Prompt, if applicable: Why are you not more likely to recommend this product to others who are exposed to?
