# Supplementary material for: Evaluating the acceptability and feasibility of new mosquito bite prevention tools in a “forest pack” to support malaria elimination in Cambodia
Source: Malar J. 2025 Nov 27;24:443. doi: 10.1186/s12936-025-05682-2 (PMC12715958; doi:10.1186/s12936-025-05682-2)
Supplement: Supplementary file 10 — Additional file10 (PDF 154 KB) [file 12936_2025_5682_MOESM10_ESM.pdf]

Appendix 3 – Key Informant Interview Guide (Implementor)

Semi-Structured Interview Guide

For this interview, we will be asking you about your experience with the BITE Forest Pack. For the purposes of this interview, we will consider the Forest Pack to include spatial repellent, topical repellent, treated clothing, and SBCC materials.

We would like to understand what aspects of the Forest Pack were feasible and sustainable over a long period (see definitions). And just as importantly, we'd like to understand what aspects of the Forest Pack were inappropriate, not useful, not practical in a real-life setting, or may not be sustainable over a long period. We are interested in how you perceive the tools from your point of view and also what you learned from end users of the tools as you were delivering the intervention.

Throughout the interview, we will discuss both the full Forest Pack (as a collection of all the tools mentioned above), and we will also ask questions about the tools individually to learn more specifically about each one.

Affiliation:
Position:
Years in position:
Gender:
Age:

Feasibility

1. Based on your experience distributing the Forest Packs, do you think this is a practical intervention for forest-goers/rangers to prevent mosquito bites? Why or why not?
2. Forest Packs were provided on a monthly basis (with treatment of clothing occurring every two months).
    a. What are your opinions on this frequency of delivery?
    b. Do you think a more frequent or less frequent delivery schedule would be more appropriate?
        i. Why would you choose that delivery schedule?
3. The goal was to provide Forest Packs to people who were most at risk of malaria infection (within a defined geographic area, based on the number of forest packs available).
    a. Do you think the Forest Packs were provided to the right people?
    b. What at risk groups were missed?

c. Were there groups of people or individuals who received the Forest Packs that were not at high risk?
d. If we were to expand the delivery of Forest Packs to an even larger population, what are some difficulties that might be encountered?
4. The delivery of the Forest Packs relied heavily on cooperation from Health Centers and VMWs.
   a. What were the benefits of this distribution method?
   b. What were the challenges with this distribution method?
   c. What would you suggest as an ideal pathway of delivery for the Forest Packs, to most effectively and efficiently reach the intended end user?

Sustainability

1. Regardless of whether you felt that the Forest Pack (and each of its products) were acceptable to you or the end user, how sustainable to do you think the delivery and use of these tools would be over a longer period of time (for example, every year during peak rainy season until malaria elimination is achieved)? *Note: Cost of the products is not a factor.*
   a. Forest Pack
      i. What might make the delivery and use of the Forest Pack sustainable or unsustainable over time?
   b. Spatial repellent
      i. What might make the delivery and use of the spatial repellent sustainable or unsustainable over time?
   c. Topical repellent
      i. What might make the delivery and use of the topical repellent sustainable or unsustainable over time?
   d. Treated clothing
      i. What might make the delivery and use of the treated clothing sustainable or unsustainable over time?
   e. SBCC materials
      i. What might make the delivery and use of the SBCC materials sustainable or unsustainable over time?
2. How do you see each of these tools potentially fitting in to the malaria elimination program in Cambodia? Can you envision how the Forest Pack, or individual products, might be used to reach elimination?
   a. Forest Pack
      i. Do you think the Forest Pack, as it was used in this study, would be useful to help reach elimination in Cambodia?
      ii. How would you envision the Forest Pack being used in the future?
   b. Spatial repellent
      i. Do you think the spatial repellent would be useful to help reach elimination in Cambodia?
      ii. How would you envision the spatial repellent being used in the future?
   c. Topical repellent

        i. Do you think the topical repellent would be useful to help reach elimination in Cambodia?
        ii. How would you envision the topical repellent being used in the future?
    d. SBCC materials
        i. Do you think the types of SBCC materials used in this study would be useful to help reach elimination in Cambodia?
        ii. What changes would you make to the SBCC materials to make them more useful in the future?
